# Polyphenol Diversity and Antioxidant Activity of European *Cistus creticus* L. (Cistaceae) Compared to Six Further, Partly Sympatric *Cistus* Species

**DOI:** 10.3390/plants10040615

**Published:** 2021-03-24

**Authors:** Brigitte Lukas, Laura Bragagna, Katharina Starzyk, Klaudia Labedz, Klaus Stolze, Johannes Novak

**Affiliations:** Institute of Animal Nutrition and Functional Plant Compounds, University of Veterinary Medicine Vienna, Veterinärplatz 1, 1210 Vienna, Austria; laura.bragagna@gmail.com (L.B.); a01005378@unet.univie.ac.at (K.S.); klaudia.lab@gmail.com (K.L.); klaus.stolze@drei.at (K.S.); Johannes.Novak@vetmeduni.ac.at (J.N.)

**Keywords:** *Cistus creticus*, Cistaceae, rockrose, HPLC, polyphenols, flavonoids, ellagitannins, antioxidant activity, chemotaxonomy

## Abstract

This investigation focused on the qualitative and quantitative composition of polyphenolic compounds of Mediterranean northern shore *Cistus creticus* and six further, partly sympatric *Cistus* species (*C. albidus*, *C. crispus, C. ladanifer*, *C. monspeliensis*, *C. parviflorus*, *C. salviifolius*). Aqueous extracts of 1153 individual plants from 13 countries were analyzed via high performance liquid chromatography (HPLC). The extracts of *C. creticus* were primarily composed of two ellagitannins (punicalagin and punicalagin gallate) and nine flavonol glycosides (myricetin and quercetin glycosides, with m-3-*O*-rhamnoside as the dominant main compound). Differences in the proportions of punicalagin derivatives and flavonol glycosides allowed the classification into two chemovariants. Plants containing punicalagin derivatives and flavonol glycosides were especially abundant in the western and central Mediterranean areas and in Cyprus. From Albania eastwards, punicalagin and punicalagin gallate were of much lesser importance and the predominant chemovariant there was a nearly pure flavonol type. With its two chemovariants, *C. creticus* takes a central position between the flavonol-rich, purple-flowered clade (besides *C. creticus*, here represented by *C. albidus* and *C. crispus*) and the more ellagitannin-rich, white- or whitish-pink-flowered clade (here represented by *C. ladanifer*, *C. monspeliensis*, *C. parviflorus* and *C. salviifolius*). The median antioxidative capacity of *C. creticus* plant material was, with 166 mg Trolox equivalents/g dry wt, about half of the antioxidative capacity of *C. ladanifer* (301 mg te/g dry wt), the species with the highest antioxidative potential.

## 1. Introduction

*Cistus* L. (Cistaceae, Malvales) comprises about 20 frutescent and suffrutescent shrub species distributed in the Mediterranean, on the Canary Islands and on Madeira. The genus is taxonomically complex, and hybridization and a high degree of morphological polymorphism complicate the determination of species boundaries. Various monographs have recognized between 16 and 28 species and have proposed conflicting intrageneric classifications. The latest taxonomic treatments based on molecular phylogenetics and pollen analyses recognized a well-supported, purple-flowered clade (equivalent to subgenus *Cistus* and including all pink-flowered *Cistus* species except for *C. parviflorus*) and a second, sometimes weakly supported white- and whitish-pink-flowered clade (comprising the two subgenera *Leucocistus* and *Halimioides* and *C. parviflorus*) [1,2]. *Cistus creticus* L. (syn. *C. incanus* auct., *C. villosus* L.) is a prominent member of the smaller, purple-flowered clade and one of the few *Cistus* species widely distributed in the eastern Mediterranean. The species seems to be a good taxonomic entity but appears to be highly variable with some geographical structuring. The high morphological variability is reflected in the plethora of scientific names given to the presently recognized *C. creticus* to distinguish the various kinds of variation. The specific name *C. creticus* seems to be widely accepted (Euro+Med PlantBase, [3]) but the putative synonyms *C. incanus* and *C. villosus* have been frequently applied in local floras and the recent literature (see list of references). Based on morphological and phytochemical characteristics, some authors recognized three subspecies, *Cistus creticus* subsp. *eriocephalus* (Viv.) Greuter and Burdet, *C. creticus* subsp. *corsicus* (Loisel.) Greuter and Burdet (both poor in essential oil) and *C. creticus* subsp. *creticus*, rich in essential oil (e.g., [4,5,6]). 

Amongst other *Cistus* species, *C. creticus* has been known as a medicinal plant since ancient times. Ladanum, exudates from the glandular hairs of the stem and leaves, or herbal infusions from leaves and upper stems have been used to heal eczema, abscesses, furuncles and diarrhea or to treat hair loss (e.g., [7,8]). Today, herbal infusions, extracts or cremes are consumed or applied for the treatment of, e.g., influenza, respiratory disorders, borrelioses or skin irritations. The diverse positive health effects observed can be attributed to a wide spectrum of secondary compounds. The essential oil of *C. creticus* is rich in labdane-type diterpenes (e.g., [5,6,9,10]). Additionally, the presence of a wide range of non-volatile compounds, mainly belonging to phenolic acids, ellagitannins and flavonols, was described [11,12,13,14]. There is thus considerable interest in this species and a vast number of publications present diverse pharmacological activities, e.g., antioxidant (e.g., [12,15]), anti-inflammatory [16], antiviral (e.g., [17,18,19]), antimicrobial (e.g., [14,20,21,22,23,24]), anticancer [25,26,27], cardiovascular protecting [28] or skin protecting (e.g., [29,30]) activities. 

Pharmacological properties and, subsequently, health benefits of plant preparations, however, are strongly dependent on their secondary compound composition. Despite the increasing knowledge of promising properties of *C. creticus* plant material, the natural variability of the responsible compounds is largely unknown. Pharmacologically, methodically or more compound characterization-orientated investigations of *C. creticus* plant material (e.g., [31,32,33]) often rely on trade samples or plant material from just one or a few pooled individual plants. In many cases, plant material used for the studies was not chemically characterized. A comprehensive overview is also hindered by the confusing taxonomic circumstances and the use of different methodical approaches that impede a comparison and summary of results. The few previous studies on natural biodiversity of *C. creticus* presented either uncharacterized NMR data [34] or were focused on the essential oil composition (e.g., [5,6,9,10]). Not much is known about abundance and intraspecific variability of non-volatile secondary compounds present in *C. creticus*. The authors of [11] investigated polyphenolic compounds of one or a few individual plants of ten different *Cistus* species native to Spain (among them *C. creticus*) and provided a first comparative overview about intrageneric diversity and species-specific characteristics of phenolic acid derivatives, ellagitannins and flavonoids. Two more publications described the flavonol and punicalagin/punicalagin gallate diversity of Cypriot *C. creticus* [35] or comparatively discussed non-volatile compounds of Sardinian *C. creticus* belonging to different subspecies [14]. The main aim of this investigation was to expand the fragmentary knowledge by comparatively characterizing flavonol compound diversity of natural populations of *C. creticus, C. albidus, C. crispus* (all three from the purple-flowered clade)*, C. ladanifer, C. monspeliensis, C. parviflorus* and *C. salviifolius* (four species of the white- and whitish-pink-flowered clade). Based on our specific experimental procedure, beside flavonols, two more compounds belonging to the ellagitannin compound family, punicalagin and punicalagin gallate, were prominently present in *C. creticus* and were included in the comparative analysis. Two additional parameters, antioxidant activity (2,2-diphenyl-1-picrylhydrazyl (DPPH)) and total phenolic content, were determined to define and comparatively describe drug activity. One thousand one hundred and fifty-three individual plants of altogether 127 populations from 13 Mediterranean countries were studied for their total phenolic content, compound composition and antioxidant activity. Additionally, 15 commercial samples were analyzed to verify the labeling and quality of *Cistus* products for medical applications. The results presented here contribute to the knowledge of chemical characteristics and inter- and intraspecific compound diversity of promising medicinal plants, may provide arguments for chemosystematic considerations as well as evidence for pharmacologists and may therewith finally help to improve drug activity and product safety.

## 2. Results

In total, one hundred and twenty-seven populations (1153 individual plants) of 8 different *Cistus* taxa were analyzed. *Cistus creticus* was paramount (72 populations, 704 individual plants), accompanied by *C. albidus* (13 populations, 131 individual plants), *C*. x *canescens* (one population, one plant), *C. crispus* (two populations, 22 individual plants), *C. ladanifer* (one population, eight plants), *C. monspeliensis* (eight populations, 89 individual plants), *C. parviflorus* (eight populations, 48 individual plants) and *C. salviifolius* (22 populations, 150 individual plants).

### 2.1. Extract Composition and Chemotypes

Chromatogram evaluation was performed at 354 nm focusing on conspicuous and recurrent peaks present in the species of main interest, *C. creticus*. Altogether, 13 major peaks (Table 1; Figure S1, Supplementary Material) representing between 55 and 100% of the total HPLC peak area (75 to 100% in *C. creticus*, 70 to 100% in *C. albidus*, 91 to 97% in *C. crispus*, 80 to 96% in *C. ladanifer*, 65 to 93% in *C. monspeliensis*, 55 to 90% in *C. parviflorus* and 60 to 100% in *C. salviifolius*; data not shown) were analyzed. Among these 13 peaks were nine flavonols, four myricetin glycosides (m-3-*O*-galactoside, m-3-*O*-glucoside, m-*O*-xyloside or m-3-*O*-arabinoside, m-3-*O*-rhamnoside) and five quercetin glycosides (q-3-*O*-rutinoside, q-3-*O*-galactoside, q-3-*O*-glucoside, q-*O*-xyloside or q-3-*O*-arabinoside, q-3-*O*-rhamnoside). Based on compound tables of the relevant literature, we initially focused on such myricetin and quercetin glycosides and were surprised by the appearance of four additional prominent peaks that eluted much earlier and exhibited distinct UV spectra. These four peaks were subsequently identified as punicalagin derivatives, more specifically two punicalagin isomers and two punicalagin gallate isomers (Table 1). 

Among the minor compounds, the presence of further myricetin glycosides (one m-*O*-rhamnoside-*O*-hexoside, one putative breakdown product of m-3-*O*-rhamnoside), quercetin glycosides (one q-pentoside, q-3-*O*-rutinoside-7-*O*-hexoside and/or q-3-*O*-(2’caffeoyl)-rutinoside and one further q-derivative) and ellagitannins (cornusiin B isomers, bis-hexahydroxydiphenoyl (HHDP)-glucose or pedunculagin, galloyl-HHDP-glucoside, probably 7-xyloside ellagic acid) was indicated (data not shown). Further minor compounds were tentatively identified as kaempferol derivatives (k-3-*O*-galactoside or -glucoside, k-diglucosides(s) or tiliroside isomers and one further putative k-derivative), flavanols ((epi)gallocatechin dimer or prodelphinidin B4, (epi)catechin, (epi)gallocatechin trimer) and a propiophenone derivative (3,4’-dihydroxypropiophenone-3-β-D-glucoside).

**Figure 1:**
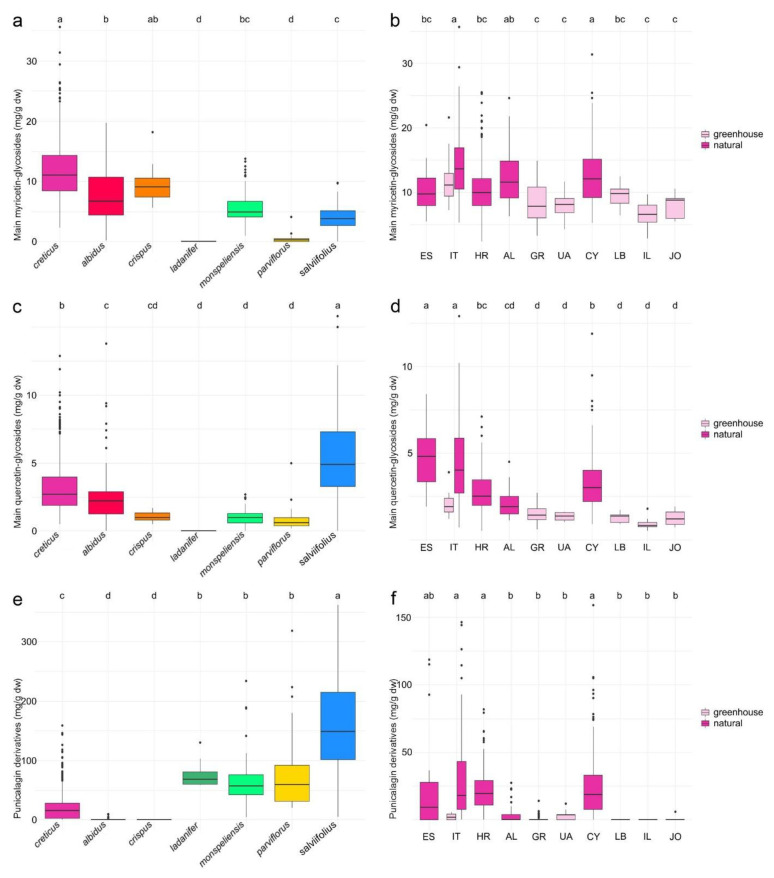
Comparison of (**a**) myricetin glycoside amounts (mg/g dry wt), (**c**) quercetin glycoside amounts (mg/g dry wt) and (**e**) amount of punicalagin derivatives (mg/g dry wt) of *C. creticus*, *C. albidus*, *C. crispus*, *C. ladanifer*, *C. monspeliensis*, *C. parviflorus* and *C. salviifolius*. Species with the same letter on top do not differ significantly from each other (groups were determined by Tukey honestly significant difference (HSD) test, alpha = 0.005). Comparison of (**b**) myricetin glycoside amounts (mg/g dry wt), (**d**) quercetin glycoside amounts (mg/g dry wt) and (**f**) ellagitannin amounts (mg/g dry wt) of *C. creticus* populations originating from Spain to Jordan. Colors determine natural (pink) or greenhouse origin (light pink) of plant material. Countries with the same letter on top do not differ significantly from each other (groups were determined by Tukey HSD test, alpha = 0.005). ES = Spain (three populations), IT = Italy (two cultivated populations and eleven natural populations), HR = Croatia (16 populations), AL = Albania (four populations), GR = Greece (four populations), UA = Ukraine (one population), CY = Cyprus (28 populations), LB = Lebanon (one population), IL = Israel (one population), JO = Jordan (one population).

To visualize the incidence and variability of the major compounds, the peak areas of the four main myricetin glycosides (m-3-*O*-galactoside, m-3-*O*-glucoside, m-*O*-xyloside or m-3-*O*-arabinoside, m-3-*O*-rhamnoside), the five main quercetin glycosides (q-3-*O*-rutinoside, q-3-*O*-galactoside, q-3-*O*-glucoside, q-*O*-xyloside or q-3-*O*-arabinoside, q-3-*O*-rhamnoside) and the two punicalagin derivatives (punicalagin and punicalagin gallate) were quantified (equivalent to the respective major compound m-3-*O*-rhamnoside, q-3-*O*-rhamnoside and punicalagin), cumulated and plotted (Figure 1).

#### 2.1.1. Myricetin Glycosides

The overall highest contents of myricetin derivatives were present in the three flavonol-rich, purple-flowering species (*C. creticus*, *C. albidus* and *C. crispus*), whereas the white (*C. ladanifer*, *C. monspeliensis*, *C. salviifolius*)- or pink (*C. parviflorus*)-flowering species exhibited lower amounts or were devoid of some of these compounds (Figure 1a). With a median content of 11 mg/g dry wt, *C. creticus* differed significantly from *C. albidus* (7 mg/g dry wt), *C. salviifolius* (4 mg/g dry wt) and the pair *C. ladanifer* and *C. parviflorus* (<LOD and 0.3 mg/g dry wt). Within the purple-flowered species, *C. crispus* (9 mg/g dry wt) could not be differentiated from *C. creticus* and *C. albidus*. *Cistus monspeliensis,* with 5 mg/g dry wt, the white-flowered species richest in myricetin glycosides, occupied a central position between *C. albidus* and *C. salviifolius*. Within the three purple-flowering species, m-3-*O*-rhamnoside was the main myricetin glycoside and main compound (up to 83% relative peak area percentage in *C. albidus*; data not shown). In rare cases, m-3-*O*-galactoside was higher than m-3-*O*-rhamnoside (up to 47% in an Albanian accession of *C. creticus*; data not shown). Within the white- or the pink-flowering species, a predominance of m-3-*O*-galactoside or m-*O*-xyloside was more frequent or the rule. Within *C. parviflorus* and *C. salviifolius*, m-3-*O*-rhamnoside was generally low or even not present (Table S1, Supplementary Material).

Within *C. creticus*, the highest amounts of myricetin glycosides were detected in Italy and Cyprus (median content of 13 and 12 mg/g dry wt, respectively; Figure 1b). Populations deriving from these two countries differed significantly from populations originating from Greece, Ukraine, Israel and Jordan grown in the greenhouse (median contents between 5 and 8 mg/g dry wt) that exhibited the overall lowest amounts of myricetin glycosides. Wild populations of Albania (12 mg/g dry wt) were not significantly different from the populations richest in myricetin glycosides. Wild populations from Spain and Croatia (both 10 mg/g dry wt) and the Lebanese greenhouse population (10 mg/g dry wt) were not significantly different from populations poor in myricetin glycosides.

#### 2.1.2. Quercetin Glycosides

With a median content of 5 mg/g dry wt, *C. salviifolius* was the species with the highest content of quercetin derivatives (Figure 1c). Significant differences were detected between *C. salviifolius*, *C. creticus* (3 mg/g dry wt), *C. albidus* (2 mg/g dry wt) and the three species comparatively low in or completely lacking quercetin glycosides (*C. ladanifer*, *C. monspeliensis* and *C. parviflorus* with contents between 0 and 1 mg/g dry wt). *Cistus crispus* (1 mg/g dry wt) took a central position between *C. albidus* and the three white-flowering species low in quercetin glycosides. The extraordinary *C. salviifolius* was the sole species with quercetin glycosides as the predominant flavonol compound family. In the flavonol-rich accessions of purple-flowering *C. creticus, C. albidus* and *C. crispus,* the contents of quercetin glycosides were usually conspicuously lower than those of the myricetin glycosides. Within *C. monspeliensis* and *C. parviflorus*, the predominance of the myricetin glycosides was not that distinctive. *Cistus ladanifer* lacked both myricetin and quercetin glycosides. In *C. creticus* and *C. albidus*, q-3-*O*-rhamnoside was often the main quercetin glycoside (up to 34% and 80% relative peak area percentage; data not shown). However, the predominance of the quercetin rhamnoside was not as pronounced as that of the myricetin rhamnoside (see above). Some accessions even showed q-3-*O*-rutinoside or q-3-*O*-galactoside as the main quercetin glycosides (about 20% of the *C. creticus* accessions, about 2% of the *C. albidus* accessions; data not shown). Within *C. crispus*, q-3-*O*-galactoside was the main quercetin glycoside. Within *C. monspeliensis*, q-3-*O*-galactoside, q-*O*-xyloside and q-3-*O*-rhamnoside were present in higher amounts. Within *C. parviflorus* and *C. salviifolius*, q-3-*O*-rhamnoside was only present in trace amounts and q-3-*O*-galactoside and q-*O*-xyloside (and/or or q-3-*O*-arabinoside) were the main quercetin glycosides (Table S1, Supplementary Material). 

Within *C. creticus*, the overall highest amounts of quercetin glycosides were present in populations of Spain and Italy (median contents of 5 and 4 mg/g dry wt; Figure 1d). Both can clearly be differentiated from populations of Cyprus (3 mg/g dry wt) and the greenhouse populations from Greece, Ukraine and the Near East (all around 1 mg/g dry wt). The populations from Croatia and Albania (both with a median content around 2 mg/g dry wt) were not clearly differentiated.

#### 2.1.3. Punicalagin Derivatives

The overall highest amounts of punicalagin and punicalagin gallate were present in the also here outstanding *C. salviifolius* (median content of 149 mg/g dry wt). *Cistus salviifolius* differed significantly from the further three white- or pink-flowering species (*C. ladanifer*, *C. monspeliensis* and *C. parviflorus,* with median contents between 57 and 68 mg/g dry wt). *Cistus salviifolius* also differed from *C. creticus* (15 mg/g dry wt) and the other two purple-flowering species (*C. albidus* and *C. crispus*), who exhibited no or solely traces of punicalagin and punicalagin gallate (Figure 1e). *Cistus creticus* was the only purple-flowered species with accessions exhibiting noteworthy amounts of punicalagin derivatives. Higher proportions of punicalagin and punicalagin gallate were usually also detected within *C. ladanifer*. *Cistus monspeliensis*, *C. parviflorus* and *C. salviifolius* were rich in punicalagin but the two punicalagin gallate isomers were not present in higher amounts (except a smaller number of single accessions from the whole distribution area) (Table S1, Supplementary Material). Beside the two punicalagin gallate peaks evaluated, however, the chromatograms of white- and whitish-pink-flowering species exhibited two additional, very conspicuous peaks with a clear punicalagin gallate signature indicating the presence of two further isomers that are specific for the white- and whitish-pink-flowered clade.

Within *C. creticus*, the highest proportions of punicalagin and punicalagin gallate were detected in the Italian, Croatian and Cypriot populations (median contents between 16 and 19 mg/g dry wt, Figure 1f). Populations from these three countries differed significantly from those of Albania, Greece, Ukraine and the Near East (all with median contents between 0 and 4 mg/g dry wt). The populations from Spain (9 mg/g dry wt) were between punicalagin derivative-rich and -poor *C. creticus* populations. Regarding the two evaluated ellagitannins, *C. creticus* was extremely variable. About 20% of the *C. creticus* samples were devoid of punicalagin and punicalagin gallate (more specifically amounts below our calculated LOD of 0.2 µg/µL). Some accessions of *C. creticus* contained lower amounts of punicalagin but no detectable amounts of punicalagin gallate, and some exhibited lower amounts of both. Single accessions reached amounts comparable to those extracted from the more ellagitannin-rich species. A high diversity was observed especially in Italy where the punicalagin derivative contents of *C. creticus* ranged from <LOD to 147 mg/g dry wt.

### 2.2. Sample Classification

**Figure 2:**
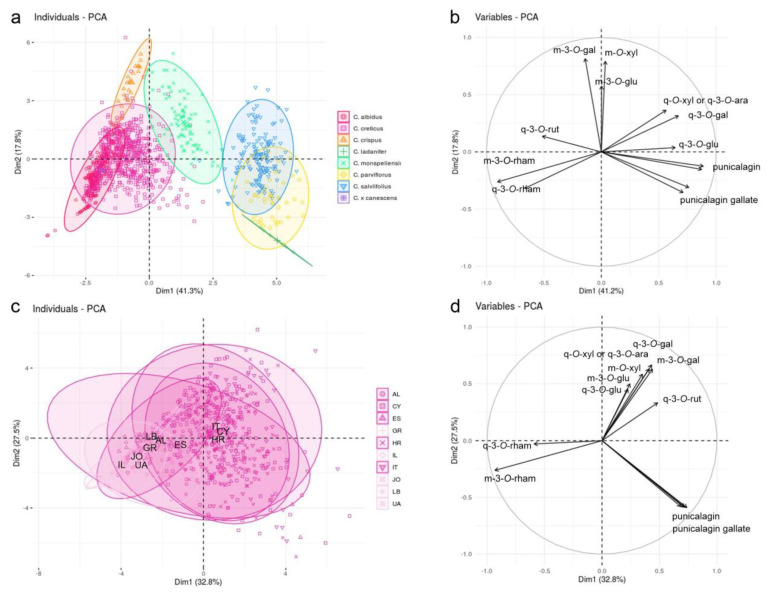
Principal component analysis (PCA) and corresponding factor loadings. (**a**) Plot of the first two dimensions (dim) from PCA performed over all seven species and the eleven evaluated compounds (relative area percent). In (**b**), the influence of each included variable is indicated. (**c**) Plot of first and second principal components from PCA performed over all sampled *C. creticus* populations (countrywise combined, group midpoints were accentuated) and the eleven main compounds used for quantification (relative area percent). (**d**) Factor loadings of the thirteen variables included. The percentage of variance explained by each dimension is indicated in parenthesis. Colors indicate either species affiliation (2a) or differentiate wild (pink) and cultivated (light pink) populations (2c). ES = Spain (three populations), IT = Italy (two cultivated populations and eleven natural populations), HR = Croatia (16 populations), AL = Albania (four populations), GR = Greece (four populations), UA = Ukraine (one population), CY = Cyprus (28 populations), LB = Lebanon (one population), IL = Israel (one population), JO = Jordan (one population).

A principal component analysis (PCA) was performed by using composition data of the 13 major peaks (relative peak area percentages; Figure 2a). The first two dimensions explained about 59% of the variance. Principal component 1 (41%) differentiated purple- from white- or whitish-pink-flowering species. The most important variables responsible for this differentiation include the two flavonols myricetin- and quercetin-3-*O*-rhamnoside as well as the four peaks representing the punicalagin derivatives (Figure 2b). Moreover, PC1 separated *C. monspeliensis* from the three other white- or pink-flowering species, with some m-glycosides as discriminating variables. Within the purple-flowered clade, principal component 2 (18% of the variation) distinguished *C. creticus* and *C. albidus* from *C. crispus*. The most influential variable here was m-3-*O*-galactoside. Within the white- and whitish-pink-flowered clade, PC2 provided only marginal distinguishing power. The separation of *C. ladanifer* individuals along a straight line reflected the complete lack of nine of the thirteen included peaks (all nine myricetin and quercetin glycosides). With a closer look at the *C. creticus* populations, no conspicuous intraspecific differentiation was obvious (Figure 2c). According to principal component 1 (33%), all the cultivated populations clustered closely together on the left margin based on the predominance of q- and m-3-*O*-rhamnoside (Figure 2d). Principal component 2 (28%) provided no noticeable discrimination between cultivated and wild populations or populations of different countries. However, the group midpoints of the geographically distant Albanian and Spanish populations clustered more closely to each other (and to the group midpoints of the cultivated populations) than to their geographically closer populations.

### 2.3. Extract Composition and Classification of Trade Samples

Fifteen trade samples of coarse-cut *Cistus* products (one trade sample was labeled as *C. creticus*, twelve as *C. incanus* and two samples as *Cistus* sp.; Table 2) were purchased from different suppliers to compare their compound composition with that of the wild and greenhouse populations. The four main myricetin glycosides summed up ranged between 1 and 10 mg/g dry wt, that of the five main quercetin glycosides between <LOD and 3 mg/g dry wt and that of the two punicalagin derivatives between <LOD and 161 mg/g dry wt (Table 2). As the peak patterns of the chromatograms of the trade samples appeared rather heterogeneous and exhibited attributes characteristic for different species, we refrained from including them as one sample group in the primary statistical analysis and plots. The heterogeneity of the trade samples was well demonstrated when included in the PCA analysis (Figure S2, Supplementary Material). Only five samples grouped within or at least close to samples of *C. creticus*, whereas the composition of the other trade samples corresponded more to that of white-flowering species (*C. monspeliensis* and *C. salviifolius*).

### 2.4. Antioxidant Activity and Total Phenolics

**Figure 3:**
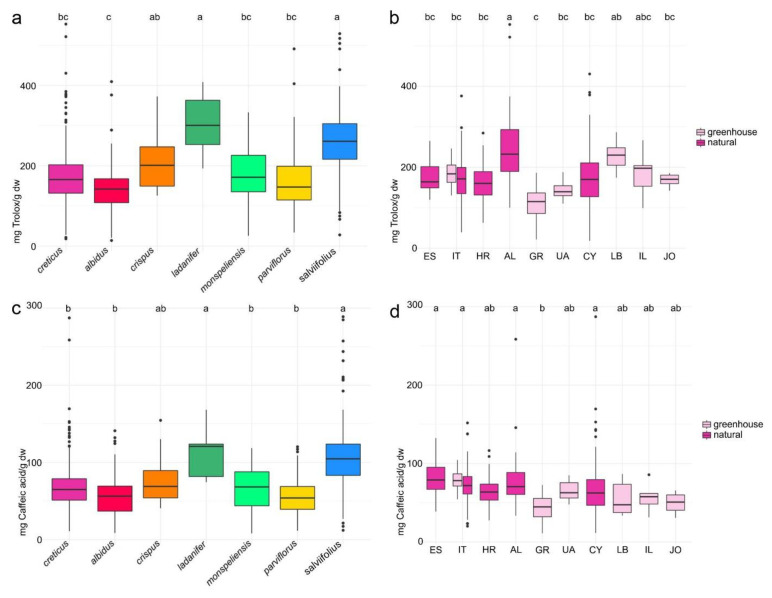
Comparison of (**a**) 2,2-diphenyl-1-picrylhydrazyl (DPPH) radical scavenging activity (mg te/g dry wt) and (**c**) total phenolic content (mg cae/g dry wt) of *C. creticus*, *C. albidus*, *C. crispus*, *C. ladanifer*, *C. monspeliensis*, *C. parviflorus* and *C. salviifolius*. Species with the same letter on top do not differ significantly from each other (groups were determined by Tukey HSD test, alpha = 0.005). Comparison of (**b**) DPPH radical scavenging activity (mg te/g dry wt) and (**d**) total phenolic content (mg cae/g dry wt) of *C. creticus* populations originating from Spain to Jordan. Colors determine either species or natural (pink) or greenhouse origin (light pink) of the analyzed plant material. Countries with the same letter on top do not differ significantly from each other (groups were determined by Tukey honestly significant difference (HSD) test, alpha = 0.005). ES = Spain (three populations), IT = Italy (two cultivated populations and eleven natural populations), HR = Croatia (16 populations), AL = Albania (four populations), GR = Greece (four populations), UA = Ukraine (one population), CY = Cyprus (28 populations), LB = Lebanon (one population), IL = Israel (one population), JO = Jordan (one population).

A DPPH radical scavenging assay was used to characterize the antioxidant activity of the *Cistus* plant samples. The highest antioxidant activities were present in *C. ladanifer* (median antioxidant capacity of 301 milligram Trolox equivalents per gram dry weight) and *C. salviifolius* (261 mg te/g dry wt; Figure 3a). Both differed significantly from *C. albidus* (142 mg te/g dry wt) with the overall lowest antioxidative activity. *Cistus crispus* (201 mg te /g dry wt) was not clearly differentiated from the two species with the highest activities. *Cistus monspeliensis* (171 mg te/g dry wt), *C. creticus* (166 mg te/g dry wt) and *C. parviflorus* (147 mg te/g dry wt) were not different from *C. albidus*, the species with the lowest antioxidant activity.

Within *C. creticus*, the highest antioxidant capacity was present in the Albanian populations (median antioxidant capacity of 232 mg te/g dry wt; Figure 3b). The populations from Albania were significantly different from greenhouse populations of closely located Greece that exhibited the lowest antioxidant activity (115 mg te/g dry wt). The Lebanese (230 mg te/g dry wt) and Israeli (198 mg te/g dry wt) populations could not be differentiated from the Albanian populations. The wild populations from Italy (174 mg te/g dry wt), Cyprus (170 mg te/g dry wt), Spain (164 mg te/g dry wt) and Croatia (160 mg te/g dry wt) as well as the greenhouse populations originating from Israel (198 mg te/g dry wt), Jordan (170 mg te/g dry wt) and Ukraine (139 mg te/g dry wt), were not significantly different from the Greek populations with the lowest antioxidative capacity. 

The total phenolic content of *Cistus* plant samples was evaluated spectrophotometrically using caffeic acid as the standard. The highest contents of phenolic compounds were again present within *C. ladanifer* (121 milligram caffeic acid equivalents per gram dry weight) and *C. salviifolius* (105 mg cae/g dry wt) that can clearly be differentiated from *C. monspeliensis* (68 mg cae/g dry wt), *C. creticus* (65 mg cae/g dry wt), *C. parviflorus* (54 mg cae/g dry wt) and *C. albidus* (56 mg cae/g dry wt; Figure 3c). *Cistus crispus* (69 mg cae/g dry wt) could not clearly be differentiated from both the species with higher and the species with comparatively low contents of total phenolics.

Within *C. creticus*, the Albanian populations (71 mg cae/g dry wt), together with Spanish (79 mg cae/g dry wt), Italian (73 mg cae/g dry wt) and Cypriot populations (62 mg cae/g dry wt; Figure 3d), exhibited the highest median contents of phenolic compounds. They were statistically different from the greenhouse populations from Greece (45 mg cae/g dry wt) with the overall lowest contents of total phenolic compounds. Populations originating from Croatia (64 mg cae/g dry wt), the Ukraine (63 mg cae/g dry wt) and the Near East (median contents of total phenolics between 47 and 58 mg cae/g dry wt) were not significantly different from both. Similar to the results from the DPPH radical scavenging assay, no significant influence of origin (from wild or cultivated populations) was detectable (data not shown). 

When comparing the plots describing DPPH and phenolic content, a high similarity between the patterns of Figure 3a,b and of Figure 3c,d became obvious, indicating a similar variability of both parameters between *Cistus* species and *C. creticus* populations from different countries. This was statistically confirmed by a strong positive correlation (*r* = 0.77) between antioxidant capacity and phenolic content (Table S2, Supplementary Material). However, there was only a positive correlation between antioxidative activity/total phenolics and the summed content of punicalagin and punicalagin gallate (*r* = 0.47/0.43) and only a rather weak positive correlation between antioxidative activity/total phenolics and the summed content of quercetin glycosides (*r* = 0.27/0.34). Myricetin glycosides do not participate much in antioxidative activity (*r* = −0.05).

## 3. Discussion

Plant material of seven *Cistus* species from 13 Mediterranean countries was sampled, aiming to provide a primary inventory of natural flavonoid variability within *C. creticus* and to allow a direct comparison of qualitative and quantitative extract composition with that of different, partly sympatric *Cistus* species.

To optimize sample preparation, initial experiments were performed to assess the influence of different sample weights and extraction times and to compare the composition of aqueous (based on the protocol of [11]) and hydromethanolic extracts (as used, e.g., by [32,39]). The different solvent extracts exhibited highly comparable peak patterns but different quantitative characteristics (Figure S3, Supplementary Material). Compared to hydromethanolic extracts (50%), pure water extracts exhibited higher amounts of the two punicalagin derivatives considered in the work (5 to 30%) and lower amounts of the main flavonoid compounds (20 to 40%). Based on the primary results, we decided on deionized water as the extraction medium because of the compositional similarity of water extracts to *Cistus* tea preparations, the most common pharmaceutical form of use. One further, pragmatic argument was the more safe and sustainable extraction procedure for the high number of samples expected. The here presented values quantifying total phenolics and antioxidant capacity might be, by trend, lower than those with hydroethanolic or hydromethanolic extracts (see also [33], who comparatively discussed characteristics of aqueous and hydroethanolic *C. incanus* extracts). 

Qualitatively, chromatograms of all the seven *Cistus* species exhibited quite stable and, for the firmly trained eye, very distinguishing peak patterns (Figure S1, Supplementary Material). Quantitatively, a wide range of variation was observed. Polyphenols are thought to be important factors for plants’ ability to cope with difficult environmental conditions and are supposed to aid their persistence in extreme habitats. As highly responsive to specific habitat conditions, polyphenolic compound levels and patterns can vary significantly within a species (e.g., [40,41,42,43]). The authors of [44] observed a moderate positive influence of temperature and solar irradiance on the total phenolic content of *C. incanus*, specifically on the content of quercetin and tannin derivatives. The authors of [45] described a suite of genes regulating flavonoid biosynthesis and transport to be largely overexpressed in sun-adapted leaves of *C. incanus* and reported a light-induced accumulation of myricetin and quercetin glycosides. Backed up by these results, it can be hypothesized that the lower compound levels of the potted populations can be primarily ascribed to latitude-related effects such as lower mean solar irradiance and mean temperatures during the year. The wild *Cistus* plants were sampled along a wide geographical gradient and from various habitats. Each individual plant was exposed to specific environmental conditions and responded to them in an individual way. To overcome this variability and to obtain statistical significance, we collected plant material from a high number of widespread populations and analyzed, in most cases, at least ten individual plants per population. To minimize effects related to plant development or season [33,44], plants exhibiting a comparable phenological stage (full to ending bloom) were harvested. Based on this carefully collected and extensive sample set, persistent overall tendencies were found. 

Extracts of *C. creticus* were usually characterized by punicalagin as the main compound in the “ellagitannin-half” and m-3-*O*-rhamnoside (in rare cases m-3-*O*-galactoside) as the main compound in the “flavonol-half” of the chromatogram. In our sample set, about 80% of the *C. creticus* plants exhibited both flavonol glycosides and punicalagin derivates. About 20 % of our *C. creticus* samples were devoid of punicalagin and punicalagin gallate (more specifically amounts <LOD of 0.2 µg/µL). This nearly pure flavonol variant was detected from Spain to the Near East, with higher abundance in the most western (37% in Spain) and more easternmost populations (40% in Albania and the Ukraine, 75% in Greece, up to 100% in populations of the Near East) and a conspicuously lesser frequency in Italy (9%), Croatia (8%) and Cyprus (12%). The rarer flavonol chemovariant relates *C. creticus* to its close relatives *C. albidus* and *C. crispus* and seems to be a specific characteristic of the purple-flowered clade, as was already postulated by [11]. Compared to *C. creticus*, the chromatogram characteristics of *C. albidus* appeared to be more stable, with the flavonol variant as the predominant one (87% of the samples) and few individual plants exhibiting comparatively small amounts of punicalagin derivatives. Within the two populations of *C. crispus*, solely the flavonol variant was detected. The frequent chemovariant characterized by the presence of flavonol glycosides and punicalagin derivatives relates purple-flowered *C. creticus* to the white- or whitish-pink-flowered species *C. ladanifer, C. monspeliensis*, *C. parviflorus* and *C. salviifolius*. Compared to *C. creticus*, the white- or whitish-pink-flowering species usually contained higher percentages and amounts of punicalagin and, in many cases, also of punicalagin gallate. Moreover, within the white- and pink-flowered species, two additional punicalagin gallate isomers were prominently present, indicating clade- and species-specific peculiarities in the respective biosynthetic pathway. In the white- or whitish-pink-flowering species, flavonol compounds were usually of less importance than in the purple-flowering ones. The one population of *C. ladanifer* completely lacked the myricetin and quercetin glycosides and both compound families were scarcely present within *C. parviflorus*. Somehow, higher percentages of flavonols were detected within *C. monspeliensis* and *C. salviifolius,* the species that was exceptional regarding its variability and partly outstanding high content of quercetin glycosides. Regarding chemotype composition, our results resembled, in principle, previous results described from single or few samples of Spanish *C. creticus*, *C. albidus*, *C. crispus*, *C. ladanifer*, *C. salviifolius* and *C. monspeliensis* [11] and Italian *C. creticus* [12,13,14,44]. One further sample of Portuguese *C. ladanifer* exhibited, contrary to [11] and our results, also smaller amounts of quercetin glycosides [36].

The first two dimensions of the principal component analysis visualize well the outcomes discussed above and earlier hypotheses that were based on the analysis of single or few extracts of ten different *Cistus* species [11]. According to their polyphenolic profiles, the purple-flowered clade (subgenus *Cistus*) could be well separated from the white- and whitish-pink-flowered clade (all four species here representing subgenus *Leucocistus*). This differentiation was mainly based on the presence and proportions of punicalagin, punicalagin gallate and m- and q-3-*O*-rhamnoside, but also further myricetin and quercetin derivatives were involved. Within the purple-flowered clade, *C. crispus* was clearly distinct, whereas *C. creticus* and *C. albidus* were not differentiated from each other. These results resembled earlier findings based on DNA sequence and pollen analysis that postulated a close evolutionary relationship of *C. creticus* and *C. albidus* with *C. crispus* as a more distantly related sister taxon [1,2]. Obviously, the separation of the purple-flowered and the white- and whitish-pink-flowered clade was not entirely perfect, mainly due to the many Italian, Croatian and Cypriot accessions of *C. creticus* that exhibited comparatively high percentages of punicalagin derivatives and tended towards subgenus *Leucocistus*. The unequal geographical distribution of plants rich in punicalagin derivatives within *C. creticus* was also lightly indicated in the PCA plots. The two group midpoints representing *C. creticus* from geographically distinct Spain and Albania, respectively, clustered more closely to each other than to the group midpoints representing the accessions from their geographically closer countries Italy and Croatia. Such an accumulation of special features in certain geographical areas of a species distribution might constitute a response to specific habitat factors (see above). However, the overall tendency of a higher frequency of punicalagin derivatives containing plants in populations originating from the mid-Mediterranean area and the minor importance of punicalagin derivatives in the continental eastern Mediterranean area were, to some degree, reflected in the greenhouse populations that were cultivated and harvested under uniform conditions. These congruent patterns would argue more for the local presence of genetic variants or for local genetic exchange. Genetic analysis run in parallel indeed detected unique DNA sequence characteristics in the Italian, Croatian and Cypriot populations (Lukas et al., unpublished). Besides the non-gradual clustering of populations related to geography, no clustering clearly related to our designation of subtaxa (subspecies or varieties) was observed. This is in accordance with [35], who analyzed flavonoid diversity of Cypriot *C. creticus* in more detail and described a conspicuous population cluster comparatively poor in flavonol glycosides that was in the most western part of the island (geographically close to or within the Polis Basin) and included populations of both *C. creticus* varieties var. *tauricus* and var. *creticus*. Based on 52 compounds, [14] could not detect significant qualitative differences in polyphenolic compound patterns of Sardinian *C. creticus* subsp. *corsicus*, subsp. *eriocephalus* and subsp. *creticus*. The subtaxon-related, intraspecific differentiation based on essential oil and NMR data [6,34] and from genetic data [6] seems not to be reflected in the polyphenolic profiles of *C. creticus*. Within the white- and whitish-pink-flowered clade, *C. monspeliensis* separated clearly, whereas the other three species were not separated. The evolutionary relationships that link white- and whitish-pink-flowered species were described to be rather complex and are still unresolved in many details [1,2]. *Cistus monspeliensis* (sect. *Ledonia* Dunal), *C. ladanifer* (sect. Ladanium (Spach.) Gren. & Godr.), *C. parviflorus* (sect. *Ledonella* Dunal) and *C. salviifolius* (sect. *Ledonia* Dunal) have been assigned to three different generic sections but none of these sections were subsequently supported by combined DNA sequence and pollen analysis [2]. The polyphenol profiles, as recorded during this investigation, did not provide distinctive signals in the case of three of the four species included. However, it must be considered that, due to the primary aim of this investigation, a higher proportion of the total HPLC peak area of the white- or whitish-pink-flowering species was not considered. The here neglected compounds may provide further distinctive signals for a clearer species discrimination. The authors of [11] defined, beside punicalagin, hexahydroxydiphenoyl-glucose, (epi)catechin, (epi)gallocatechin and an (epi)catechin-(epi)gallocatechin dimer as compounds with high discriminating power.

The overall highest antioxidative activities were observed in two species of the white- and whitish-pink-flowered clade, *C. ladanifer* (mean value of 303 mg te/g dry wt) and *C. salviifolius* (264 mg te/g dry wt). As expected, the antioxidative capacity correlated strongly with the total phenolic content but there was solely a moderate (punicalagin derivatives), weak (quercetin glycosides) or even no (myricetin glycosides) correlation between antioxidant activity and quantified contents of main compounds and main compound families. In particular, the weak or lacking statistical correlation between antioxidative activity and myricetin or quercetin glycosides was rather surprising. Myricetin, quercetin and some of their glycosides, especially their rhamnosides, were described to be powerful antioxidants, with an antioxidant activity similar to or slightly weaker than that of vitamin E [37,46]. However, when comparing the plots of total, myricetin, quercetin and punicalagin derivative contents of the different species with the plot visualizing their antioxidative capacity, the patterns are obviously not congruent. This was especially obvious within *C. creticus*. Although strikingly poorer in these compounds, the antioxidative capacity of plant material from some cultivated *C. creticus* populations was comparable to that of many natural populations. These findings imply that, beside the recorded punicalagin derivatives and flavonol glycosides, further components (possibly not that sensitive to certain growing conditions) must be significantly involved in the antioxidative capacity of *Cistus* plant material. Such candidate compounds would be, e.g., hexahydroxydiphenoyl-glucose, gallocatechin, gallic acid and catechin that were, besides m-3-*O*-rhamnoside, identified as compounds with stronger antioxidant activity in *C. incanus* herbal tea infusions [36].

Within the purple-flowered clade, plant material of *C. crispus* (206 mg te/g dry wt) and plant material of *C. albidus* (142 mg te/g dry wt) exhibited a slightly higher and a slightly lower antioxidative activity, respectively, than that of *C. creticus* (170 mg te/g dry wt). Compared to the white- or whitish-pink-flowered species, the antioxidative capacity of *C. creticus* plant material was about half of that of *C. ladanifer*, about two thirds of that of *C. salviifolius* or close to that of *C. monspeliensis* (175 mg te/g dry wt) and *C. parviflorus* (169 mg te/g dry wt). Direct comparisons of phenolic contents and antioxidant activity with results of previous investigations of *Cistus* plant material were difficult as various experimental conditions and different modes of result expression were used. The total phenolic contents determined in the course of this investigation seem to be somehow higher than those published by [47] (about 55 mg gallic acid equivalents (gae)/g dry wt in ethanolic extracts of Tunisian *C. monspeliensis* and *C. salviifolius*), seem to somehow resemble those of [32] (about 65 mg gae/g dry wt in an aqueous extract of Syrian *C. creticus*, about 70 mg gae/g dry wt in an aqueous extract of *C. salviifolius*) or seem to be somehow lower than those predicted by [48] (about 250 mg gae/g dry wt in an ethanolic extract of a Portuguese *C. ladanifer* sample), [33] (up to 115 mg gae/g dry wt in ethanolic extracts of a Bulgarian *C. creticus* sample), [49] (about 500 mg gae/g dry wt in an ethanolic extract of a Tunisian *C. salviifolius* leaf sample) or [39] (about 408 mg gae/g dry wt or 335 mg gae/g dry wt in aqueous extracts of Moroccan *C. salviifolius* and *C. monspeliensis*). When comparing *C. creticus* to prominent aromatic plants of the Lamiaceae family that were previously studied in our lab by the same quantification method used here, the antioxidative activity of *C. creticus* was higher than that of *Salvia officinalis* [50] or *Thymus vulgaris* L. [51]. However, the total phenolic content of *C. creticus* was comparable to that of *Salvia officinalis* [50] and slightly higher than that of *Thymus vulgaris* L. [51], indicating specific compounds of higher activity in *Cistus*.

In recent years, *Cistus* plant material and *Cistus* preparations have been increasingly used for diverse medicinal purposes. However, up to date, no *Cistus* monograph is available in the European Pharmacopoeia [52] to define target species and minimum quality parameters. Despite its long traditional use in some South European countries, *Cistus* is subject to the “novel food regulation” (EU 2015/2283). A certain variety locally native to northern Greece, *Cistus incanus* L. Pandalis herba, has been registered in the category herbal infusions [53]. Apart from pharmacies, different *Cistus* plant materials are often offered as a “bath additive”. In the face of lacking guidelines for drug quality requirements and confusion caused by the inconsistent categorization of *Cistus* plant material, it is doubtful that consumers can rely on a constant active substance content and a consistent quality and purity of their health remedies. To validate the polyphenolic content and composition of currently available commercial products, 15 trade samples of coarse-cut *Cistus* plant material from different trademarks, pharmacies and health retailers were included in our analysis. Twelve of these trade samples were originally labeled as *C. incanus* and one as *C. creticus*, and two further *Cistus* herbal “teas” had no species designation on the label. The content of water-soluble compounds of the trade samples varied highly, from nearly zero to well comparable with that of plant material collected during this investigation. This striking variability in polyphenolic compound levels might reflect differences in individual sample composition, age or sampling and storage conditions of trade batches. The authors of [31] revealed that the wooden fraction of trade samples contained only small amounts of polyphenols compared to the leafy fraction. There was also high variability concerning the qualitative composition of the aqueous extracts, what was immediately visible from the characteristic peak patterns of their chromatograms. The principal component analysis then placed six of the trade samples within or close to the *C. creticus/C. albidus* cluster, whereas the other nine samples seemed to resemble more the typical qualitative characteristics of white-flowering species. Small white flower pieces present in some of these trade samples subsequently confirmed our results. These outcomes led to the conclusion that (currently) trade batches of *Cistus* plant material can differ highly in quality and are almost certainly not designated correctly or are at least admixtures of *C. creticus* and different *Cistus* species. These findings should be considered when postulating, assigning or comparing pharmaceutical effects based on results gained from the analysis of few *Cistus* trade sample preparations.

## 4. Materials and Methods

### 4.1. Plant Material

**Figure 4:**
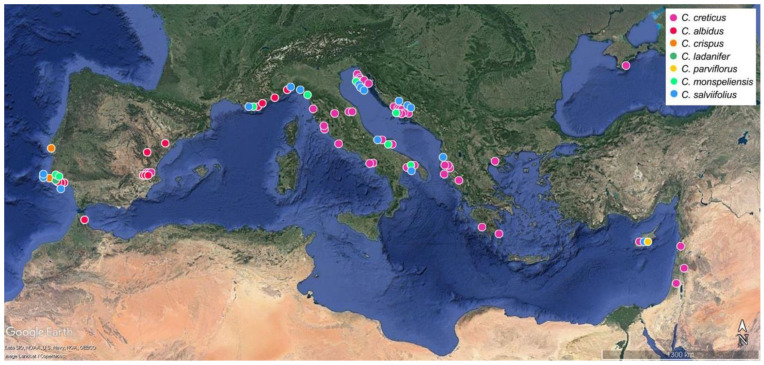
Geographical origin of the *Cistus* populations analyzed (distribution map was compiled with Google Earth (https://www.google.com/earth/download/ (accessed on 12 March 2020)). The three marks in Cyprus represent the 29 populations of *C. creticus*, eight populations of *C. parviflorus* and seven populations of *C. salviifolius* sampled (mainly) in Southern Cyprus. Geographical coordinates are summarized in Table S1, Supplementary Material.

One hundred and twenty-seven populations and 1153 individual plants of *C. creticus* L. (72 populations, 704 individual plants), *C. albidus* L. (13 populations, 131 individual plants), *C*. x *canescens* Sweet (one population, one plant), *C. crispus* L. (two populations, 22 individual plants), *C. ladanifer* L. (one population of eight plants), *C. monspeliensis* L. (eight populations, 89 individual plants), *C. parviflorus* Lam. (eight populations, 48 individual plants) and *C. salviifolius* L. (22 populations, 150 individual plants) and 15 trade samples from different suppliers were analyzed for this investigation. The main part of the plant material was collected in the wild from native populations in Albania, Croatia, Cyprus, France, Italy, Portugal and Spain, in autumn 2016 (Albania) or the late spring of 2017 and 2018. Except for the five Albanian populations and two Portuguese populations of *C. crispus,* the bigger part of the wild plants was harvested at a comparable phenological stage (beginning to end of bloom). The second part of the analyzed plant material was from potted *C. creticus* (ten populations) and *C. albidus* (five populations) plants grown in the greenhouse (greenhouse cultivation in winter, open land cultivation from early spring to late autumn) at the University of Veterinary Medicine Vienna. Seeds for the greenhouse-grown plants were obtained from the Millenium Seedbank (Royal Botanic Gardens Kew) and the Seed Bank Berlin Dahlem. The cultivated plants were in full bloom when they were harvested in their second vegetation period (early summer 2018). Geographical locations of populations can be seen in Figure 4, geographical coordinates of the native populations and seedbank accession numbers are summarized in Table S1 (Supplementary Material) and trade samples are characterized in Table 2. The included picture gallery (Supplementary Material) offers a view of selected natural populations and potted plants. All plant material of wild populations was sampled in accordance with the guidelines of the Nagoya Protocol (https://www.cbd.int/abs/text (accessed on 15 September 2016)). Species were identified by following keys of the local floras (references are provided in Table S1, Supplementary Material). Voucher specimens of wild and cultivated populations, currently kept at the herbarium of the Institute for Animal Nutrition and Functional Plant Compounds, University of Veterinary Medicine Vienna, will be submitted to the Herbarium of the Institute of Botany, University of Vienna (WU). Copyright of pictures in the graphical abstract belongs to Johannes Novak, Corinna Schmiderer, Martina Pettighofer and, in the case of *C. crispus*, Willem van Kruijsbergen (Saxifraga Foundation; http://www.freenatureimages.eu (accessed on 15 October 2020).

### 4.2. Sampling Procedure and Handling of Plant Material

From each individual sampled plant, one representative branch was collected from the canopy top. The plant material was either air dried (wild populations) or dried in a drying cabinet (30 °C, greenhouse populations). The dry plant material was then kept in cartons at room temperature. For analysis, all leaves of a branch were separated from the stems. Stems and, when present, flowers and early fruits were removed. Immediately before extraction, a representative portion of the roughly crushed leaves was ground to a fine powder by using a ball mill (Pulverisette, Fritsch, Germany).

### 4.3. Extractions

One hundred and fifty mg of finely grounded plant material was extracted with eight ml milli-Q water at 60 °C, for 120 min in a shaking water bath (based on the protocol of [11]). The filtered extracts were aliquoted in HPLC vials (for HPLC analysis) and in 1.5 mL Eppendorf tubes (for analysis of DPPH and total phenolics). The aliquots were kept at −20 °C until analysis.

### 4.4. HPLC and HPLC-MS Analysis

HPLC analyses were performed using a Shimadzu Nexera XR chromatograph (Shimadzu, Austria) equipped with a controller (CBM-20A), a degasser (DGU-20A5R), a quaternary pump (LC-20ADXR), an autosampler (SIL-20AXR), a column oven (CTO-20AC) and a photodiode array detector (SPD-M20A). The software package LabSolutions 5.82 (Shimadzu, Austria) was used for data collection and processing. Separations were performed on a XBridge™ Shield RP18 column (3.5 µm, 4,6 × 150 mm; Waters, Austria) equipped with a C18 guard column (ODS Octadecyl, 4 mm × 0.3 mm, Phenomenex, Germany). A linear gradient elution was carried out at a flow rate of 1 mL/min and an oven temperature of 25 °C using acetonitrile (Carl Roth, Germany; solvent A) and 2% acetic acid (Carl Roth, Germany; solvent B). The following gradient was used: min 0–38, 6–17% A in B (linear gradient); min 38–53, 17–20% A in B (linear gradient); min 53–58, 100% A (isocratic); min 58–65, 0–6% A in B (linear gradient). Injection volume was 20 µL. Peak detection was performed at 354 nm, close to the UV/Vis absorption maximum of many flavonoids.

After visual inspection of the first 30 chromatograms of Cypriot *C. creticus*, 13 prominent and/or recurrent peaks were defined and subsequently evaluated (Table 1 and Figure S1, Supplementary Material). Identification and verification of trans-species occurrence of these prominent compounds were conducted via literature data (references are provided in Table 1), by comparing retention times and UV spectra to those of available reference chromatography standards (punicalagin, myricetin-3-*O*-rhamnoside, quercetin-3-*O*-rutinoside, quercetin-3-*O*-galactoside, quercetin-3-*O*-glucoside, quercetin-3-*O*-rhamnoside; all from Phytolab, Germany) and by comparative runs of selected accessions of the seven *Cistus* species, commercial standards and characterized green tea samples on a HPLC-MS (Waters, Austria) (Table 1). The HPLC-MS was equipped with a separation module (Waters 2695), a photodiode array detector (Waters 996) and a mass spectrometer (Waters Micromass Quattro micro^TM^). Various runs using alternative columns, solvents/gradients and scan modes were performed and comparatively analyzed to trace selected (mass) components and to verify the presence or composition of multiple peaks. The ESI source was operated in negative mode using the following conditions: capillary voltage 2.5 kV, cone voltage 35 V, extractor 3 V, RF lens 0 V, source temperature 150 °C, desolvation temperature 350 °C. Nitrogen was set at 600 L/min. The unambiguous identification of smaller or minor peaks, however, was finally hindered by the combination of low signal strength and slight retention time shifts and pattern shifts when comparing HPLC and HPLC-MS chromatograms. 

The quantification of punicalagin and punicalagin gallate as well as that of main myricetin and quercetin glycosides was conducted by comparison with external standards of punicalagin (y = 3345451x, r^2^ = 0.995; LOD = 0.189 µg/µL, LOQ = 0.631 µg/µL; quantification of punicalagin gallate was performed equivalent to punicalagin), myricetin-3-*O*-rhamnoside (y = 36317204 x, r^2^ = 0.998; LOD = 0.0199 µg/µL, LOQ = 0.066 µg/µL; quantification of m-3-*O*-galactoside, m-3-*O*-glucoside and m-*O*-xyloside was performed equivalent to m-3-*O*-rhamnoside) and quercetin-3-*O*-rhamnoside (y = 42551527 x, r^2^ = 0.999; LOD = 0.009 µg/µL, LOQ = 0.032 µg/µL; quantification of q-3-*O*-rutinoside, q-3-*O*-galactoside, q-3-*O*-glucoside and q-*O*-xyloside or q-3-*O*-arabinoside was performed equivalent to q-3-*O*-rhamnoside). Quantification was expressed as milligram per gram dry weight (mg/g dry wt). 

### 4.5. Total Phenolics

The total phenolic content was evaluated as described in [50], with small modifications. The water extracts were diluted with milli-Q water (1:10). Ten µL of the dilution was mixed with 100 µl of milli-Q water and 5 µL of Folin–Ciocalteu’s phenol reagent (Merck, Germany) in a microplate well. The mixture was kept at room temperature for 3 min and then 10 µL of Na_2_CO_3_ solution (Carl Roth, Germany; 35 g in 100 mL milli-Q water) and 125 µL of milli-Q water were added. After 60 min of incubation in the dark, the absorbance at 750 nm was measured using a microplate reader (i-Mark, Bio-Rad, Austria). Caffeic acid (Sigma-Aldrich, Austria; 10 mg in 100 mL milli-Q water) was used as standard. A blank was used to correct the readings. Calibration points and samples were pipetted and measured as quadruplicates. The results were expressed as milligram of caffeic acid equivalents per gram dry weight (mg cae/g dry wt).

### 4.6. DPPH Radical Scavenging Activity

The DPPH radical scavenging activity was evaluated according to [50]. The water extracts were diluted with milli-Q water (1:10). One hundred and five µL of the dilution was then mixed with 95 µL methanol (Carl Roth, Germany) and 100 µL of solution (2,2-diphenyl-1-picrylhydrazyl, Sigma-Aldrich, Germany; 0.0038 g in 25 mL methanol). After 30 min incubation in the dark at room temperature, the absorbance of the reaction mixture was measured at 490 nm using a microplate reader (i-mark, Bio-Rad, Austria). Trolox (6-hydroxy-2,5,7,8-tetramethylchroman-2-carboxylic acid, Sigma-Aldrich, Austria; 0.0063 g in 10 mL pure ethanol) was used as standard. A blank was used to correct all readings. Calibration points and samples were pipetted and measured as quadruplicates. The results were expressed in milligram Trolox equivalents per gram dry weight (mg te/g dry wt).

### 4.7. Statistics

Statistical analyses (basic statistical parameters, correlations, Analysis of Variance (ANOVA), Tukey honestly significant difference (HSD) test, principal component analysis (PCA)) were performed and visualized by using R 3.5.2 [54] and the packages agricolae, corrplot, dplyr, factoextra, FactoMiner, ggplot2, ggpubr, ggsci, Hmisc, multcompView, PerformanceAnalytics, readxl, tidyverse and RColorBrewer. 

## 5. Conclusions

*Cistus creticus* exhibited an impressive diversity in total content of water-soluble compounds and in contents of punicalagin derivatives and flavonol glycosides. Two chemovariants based on the presence/absence of punicalagin derivatives were identified: a more frequent one containing punicalagin derivatives and a rarer one without punicalagin derivatives. Punicalagin derivatives containing plants accumulated regionally in the western and especially the mid-Mediterranean areas and in Cyprus. In populations of the eastern Mediterranean area, punicalagin and punicalagin gallate were (almost) absent. Beside this large-scale pattern, there was no obvious correlation between polyphenolic profiles and small-scale morphological diversity supporting a classification of *C. creticus* variants to subspecies or varieties. Natural and cultivated *C. creticus* populations differed significantly in flavonol glycoside and punicalagin derivative contents but not in total phenolic content and antioxidative capacity. Compared to the *Cistus* species with the overall highest antioxidative capacity, *C. ladanifer,* the antioxidative capacity of *C. creticus* was approximately half. Compared to two Lamiaceae species often declared as medicinal plants with high antioxidative capacity, *Salvia officinalis* and *Thymus vulgaris*, the antioxidative capacity of *C. creticus* was higher.

The specific polyphenolic compound composition of *Cistus* species seems to be related to evolutionary events. Based on relative percentages of punicalagin derivatives and the main flavonol glycosides, purple-flowered and white- and whitish-pink-flowered clades could principally be well separated. Within the purple-flowering subgenus *Cistus, C. crispus* differentiated clearly from the strongly overlapping clusters of *C. creticus* and *C. albidus*. More punicalagin derivative-rich plants of *C. creticus* segregated towards subgenus *Leucocistus* and might indicate an evolutionary event differentiating western/mid-Mediterranean populations and eastern Mediterranean populations of *C. creticus*. Within subgenus *Leucocistus*, *C. monspeliensis* separated clearly, whereas the other three species did not differentiate. 

## Figures and Tables

**Table 1:** HPLC retention times (Rt) of the 13 evaluated peaks, mass data (base ions at negative mode ([M-H]^−^), main fragment ions (MS/MS)), peak identification (M = myricetin, Q = quercetin) and relevance of compounds within aqueous extracts of *C. creticus* (range of relative area percentages at 354 nm). ^1^ Highlights compounds that were identified via reference compounds; ^2^ highlights peaks that were shown to be double peaks in a part of the accessions (characteristics of the overlain peak are indicated below the respective line). Identification literature [11,12,13,26,31,36,37,38].

Peak	Rt (min)	[M-H]^−^ (*m/z*)	MS/MS (*m/z*)	Proposed Compound	Literature	Rel. Area %
1	10.1	1083	301/541	Punicalagin, isomer 1 ^1^	[11,13,26,31,36]	0–16%
2	14.4	1083	301/541	Punicalagin, isomer 2 ^1^	[11,13,26,31,36]	0–19%
3	14.8	1251	541/603	Punicalagin gallate, isomer 1	[11,26,31,36]	0–10%
4	22.1	1251	541/603	Punicalagin gallate, isomer 2	[11,26,31,36]	0–14%
5	32.1	479	316	M-3-*O*-galactoside	[11,12,26,31,37,38]	0–47%
6	32.8	479	316	M-3-*O*-glucoside	[11,12,13,26,31,37,38]	0–6%
7	37.3	449	316	M-*O*-xyloside and/or m-3-*O*-arabinoside ^2^	[26,31,37,38]	0–50%
8	38.4	463	316	M-3-*O*-rhamnoside (myricitrin) ^1^	[11,12,13,26,31,37,38]	5–83%
9	39.3	609	301	Q-3-*O*-rutinoside (rutin) ^1, 2^	[11,12,13,26,31,37,38]	0–17%
		521	316	Putative m-derivative, ev. an artifact		
10	40.0	463	301	Q-3-*O*-galactoside (hyperoside) ^1^	[26,31,37,38]	0–12%
11	40.8	463	301	Q-3-*O*-glucoside (isoquercetin) ^1^	[11,13,26,31,36,37,38]	0–3%
12	46.0	433	301	Q-*O*-xyloside and/or q-3-*O*-arabinoside ^2^	[11,12,13,26,31,36,37,38]	0–9%
13	48.9	447	301	Q-3-*O*-rhamnoside (quercitrin) ^1^	[11,12,13,26,31,37,38]	0–35%

**Table 2:** Designation and geographical origin (when indicated) of the 15 commercial trade samples purchased from different pharmacies ^1^ or health retailers ^2^ (kbA = controlled organic cultivation) and their contents of punicalagin derivatives and myricetin and quercetin glycosides (mg/g dry weight).

Sample	Declaration	Origin	P-Derivatives	M-Glycosides	Q-Glycosides	Tentative Identification
			(mg/g dry wt)	(mg/g dry wt)	(mg/g dry wt)	(via HPLC profile)
CHP01 ^1^	*Cistus* sp.	TR	161.2	4.8	2.4	White-flowered species
CHP02 ^1^	*C. incanus* L.	GR	<LOD	4.0	0.8	*C. creticus*
CHP03 ^1^	*C. incanus*	-	16.8	10.0	2.5	*C. creticus*
CHP04 ^1^	*C. incanus* L.	GR	<LOD	0.7	0.2	*C. creticus*
CHP05 ^2^	*C. incanus*	-	81.1	4.1	1.5	White-flowered species
CHP06 ^2^	*C. incanus* ^kbA^	-	33.1	0.5	0.2	White-flowered species
CHP07 ^2^	*C. incanus* ^kbA^	CY	85.3	8.4	2.3	*C. creticus*
CHP08 ^2^	*C. incanus*	TR	22.1	8.6	2.0	*C. creticus* (adulterated?)
CHP09 ^2^	*C. incanus*	-	50.7	5.9	1.7	White-flowered species
CHP10 ^2^	*C. incanus*	-	61.5	4.0	1.3	White-flowered species
CHP11 ^2^	*C. incanus*	-	79.6	3.5	1.1	White-flowered species
CHP12 ^2^	*C. creticus*	-	123.3	3.3	3.0	White-flowered species
CHP13 ^2^	*C. incanus* ^kbA^	TR	66.0	3.2	1.4	White-flowered species
CHP14 ^2^	*C. incanus*	-	82.9	5.1	2.7	White-flowered species
CHP15 ^2^	*Cistus* sp.	TR	29.4	6.5	1.5	*C. creticus* (adulterated?)

## Data Availability

Data are contained within the article.

## Supplementary Materials

The following are available online at https://www.mdpi.com/2223-7747/10/4/615/s1, Figure S1: (a) Example chromatogram of Cypriot *C. creticus* recorded at 354 nm. Red marks refer to the retention times of the 13 peaks defined after initial inspection of representative chromatograms. The peak numbers indicated in the graph correspond to the peak numbers and compounds listed in Table 1 and Table S1. (b) Direct comparison of representative example chromatograms of *C. creticus* (SRC580, cre), *C. albidus* (SRC71, alb), *C. crispus* (SRC135, cri), *C. ladanifer* (SRC59, lad), *C. monspeliensis* (SRC115, mon), *C. parviflorus* (SRC595, par) and *C. salviifolius* (SRC96, sal). Identification of the quantified main compounds is indicated., Figure S2: Principal Component Analysis—plot of the first two dimensions from PCA performed over all seven species and the eleven evaluated main compounds (relative area percent). Black squares indicate the position of the 15 trade samples. Figure S3: HPLC chromatograms (354 nm) of SRC170 and SRC204 (*C. creticus*, Cyrus), comparison of hydromethanolic extract (50:50; black line) and pure water extract (pink line). Table S1 (sheet 1): Geographical origin of natural and cultivated *Cistus* populations, collection details, number of plants analysed, population mean values and standard deviations of quantified main components, total phenolics and antioxidative activity. Table S1 (sheet 2): Species minimum values, maximum values, mean values and standard deviations of main components, total phenolics and antioxidant activity. Table S2: Statistical correlation between total phenolics (mg cae/g dry wt), antioxidant activity (mg te/g dry wt) and sums of punicalagin derivatives, myricetin- and quercetin-glycosides (mg/g dry wt). Picture gallery: View of selected natural *C. creticus* populations and potted *Cistus* plants.
