# Supplementary material for: Polyphenol Diversity and Antioxidant Activity of European Cistus creticus L. (Cistaceae) Compared to Six Further, Partly Sympatric Cistus Species
Source: Plants (Basel). 2021 Mar 24;10(4):615. doi: 10.3390/plants10040615 (PMC8063833; doi:10.3390/plants10040615)

Picture Gallery

Pictures provided from Brigitte Lukas, Johannes Novak, Corinna Schmiderer

C. albidus, wild populations in Portugal

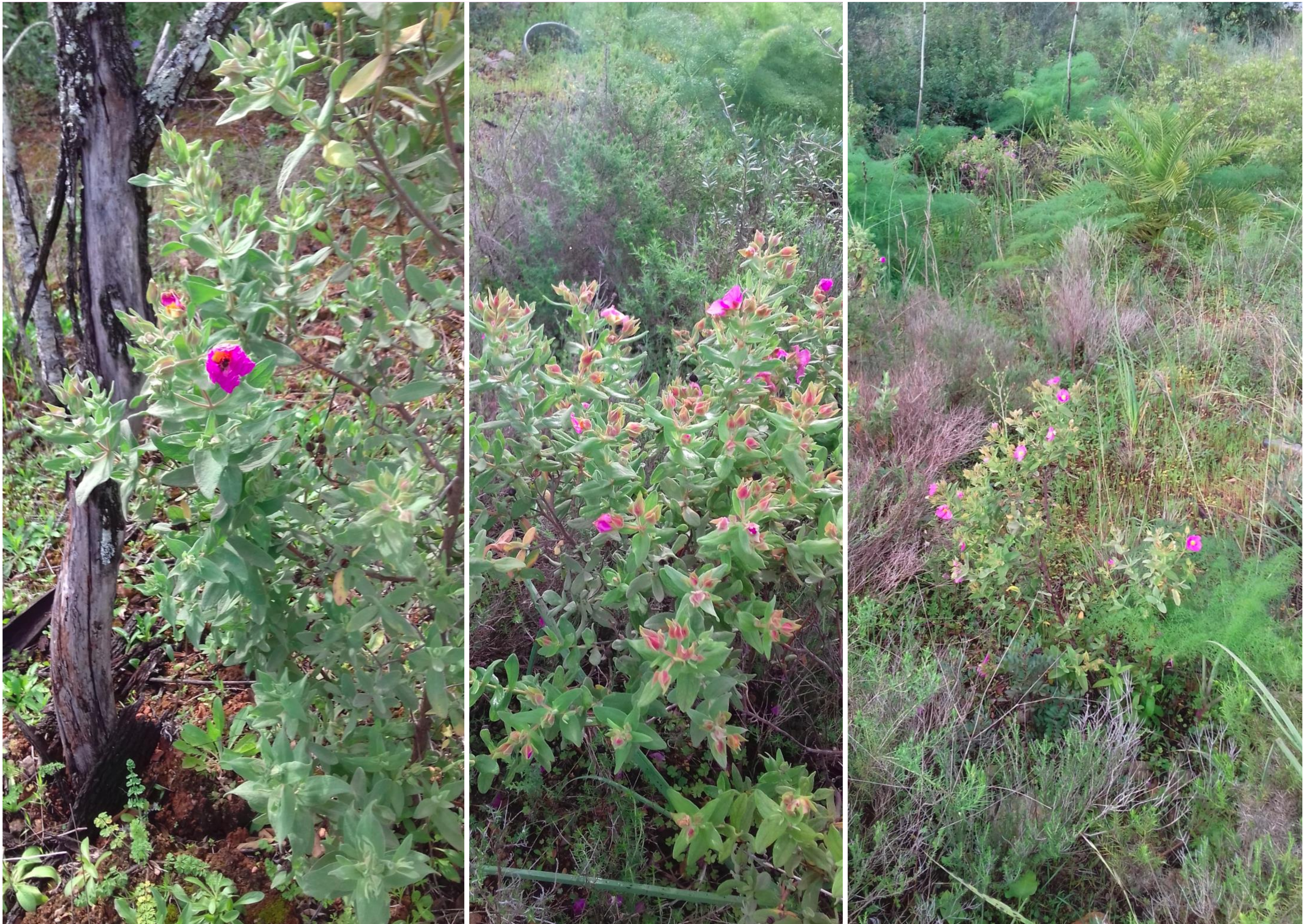

SC7, *C. albidus*, Spain

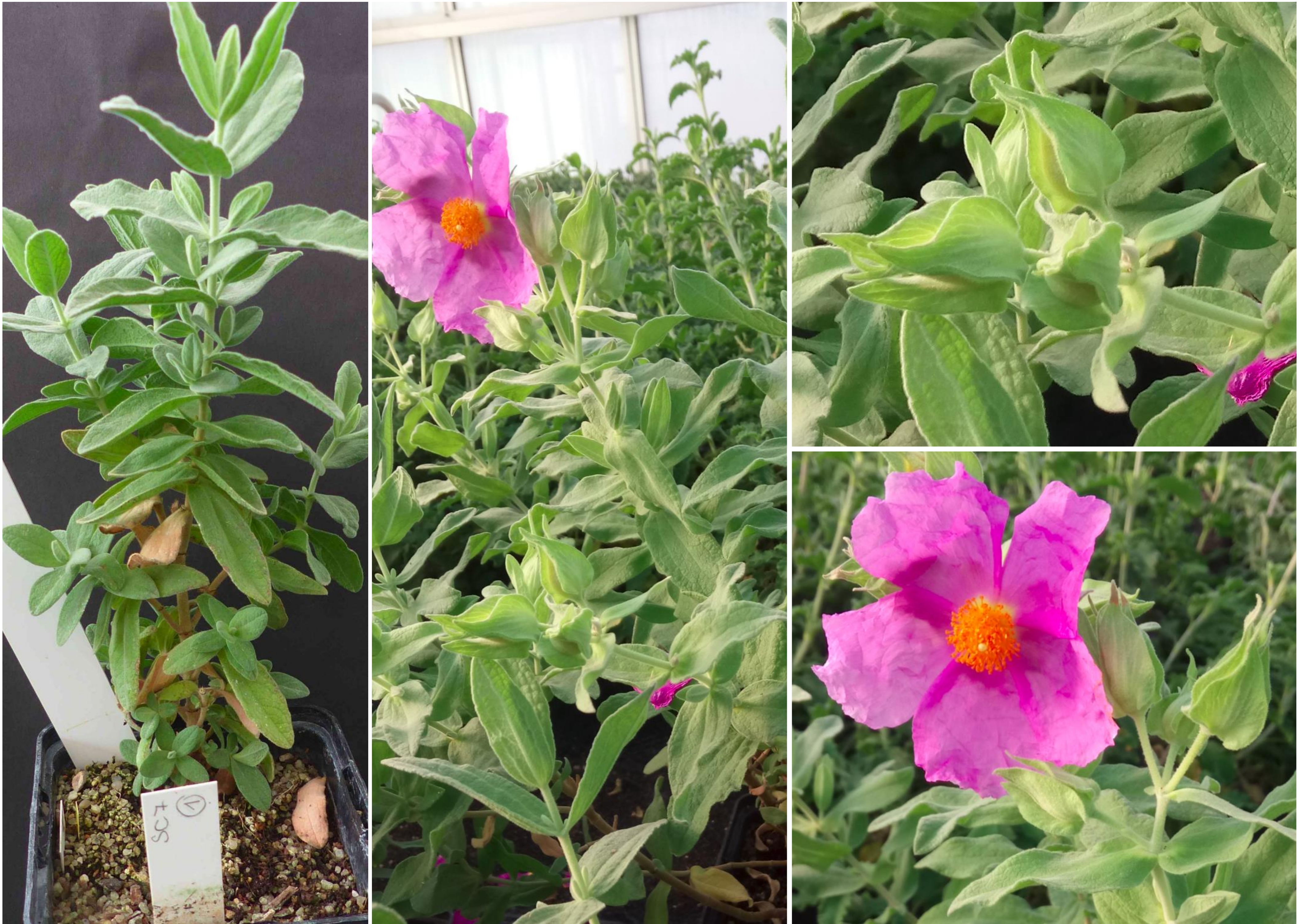

SC10, *C. albidus*, Spain

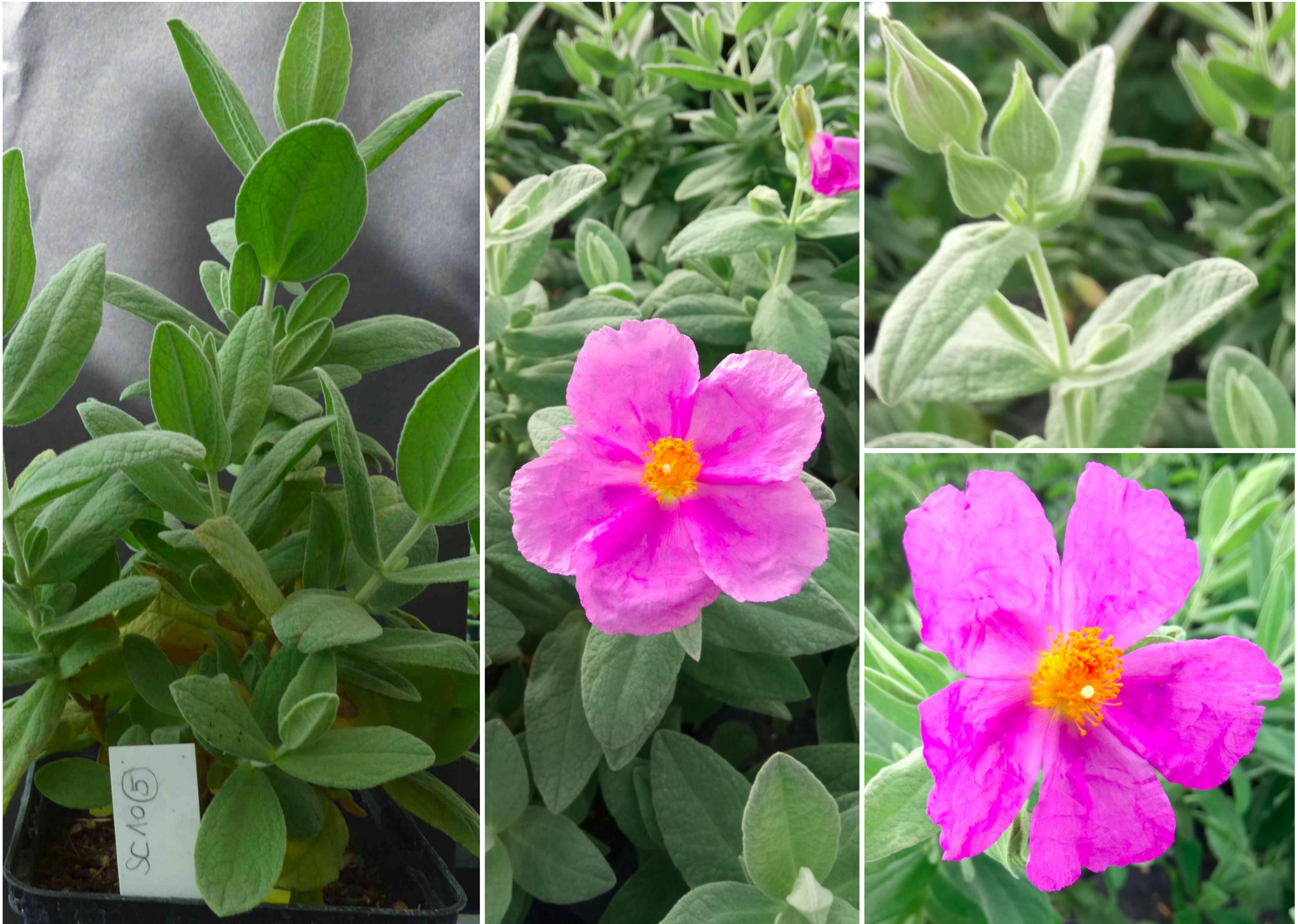

C. creticus, wild populations in Italy

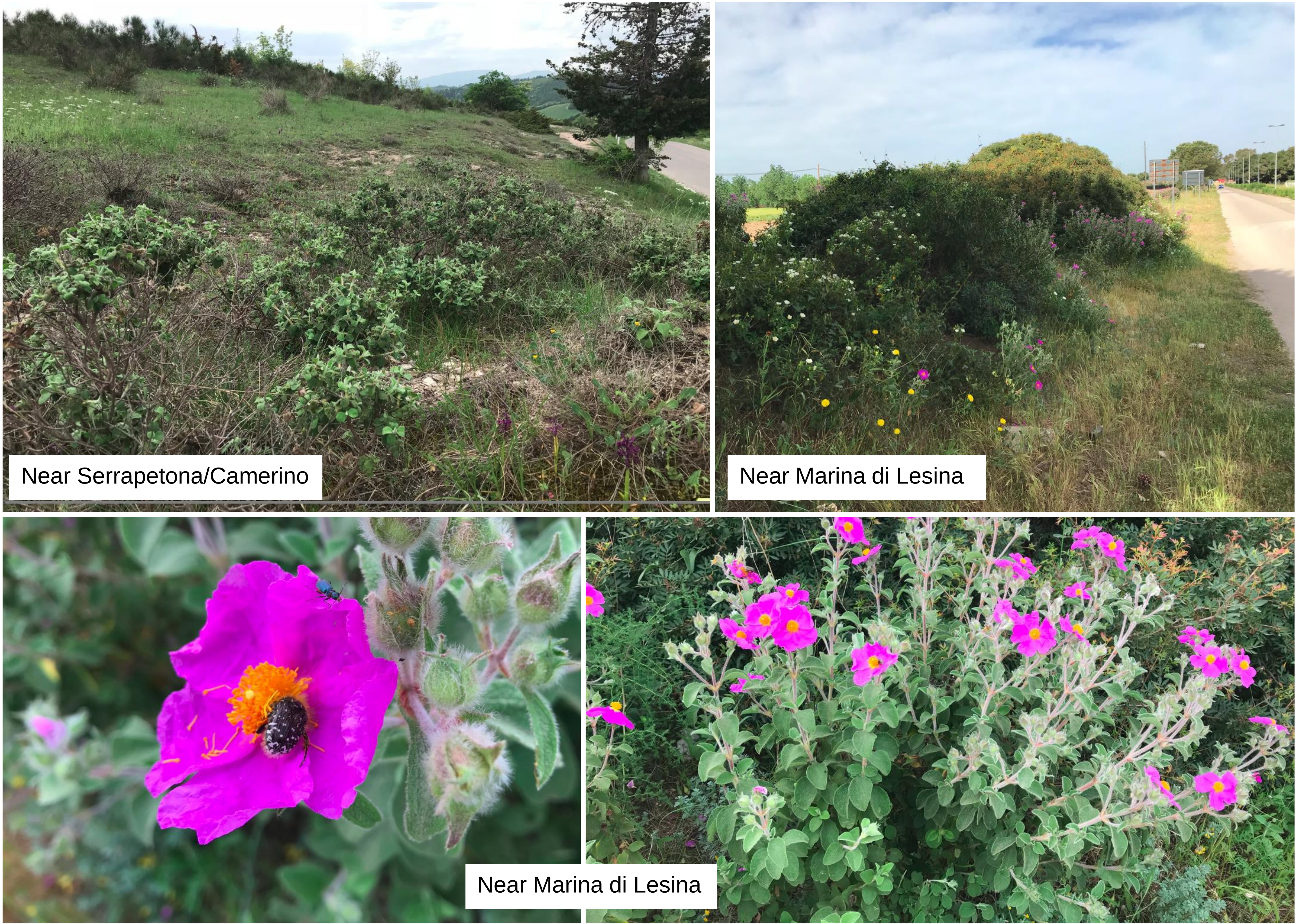

C. creticus, wild populations in Italy

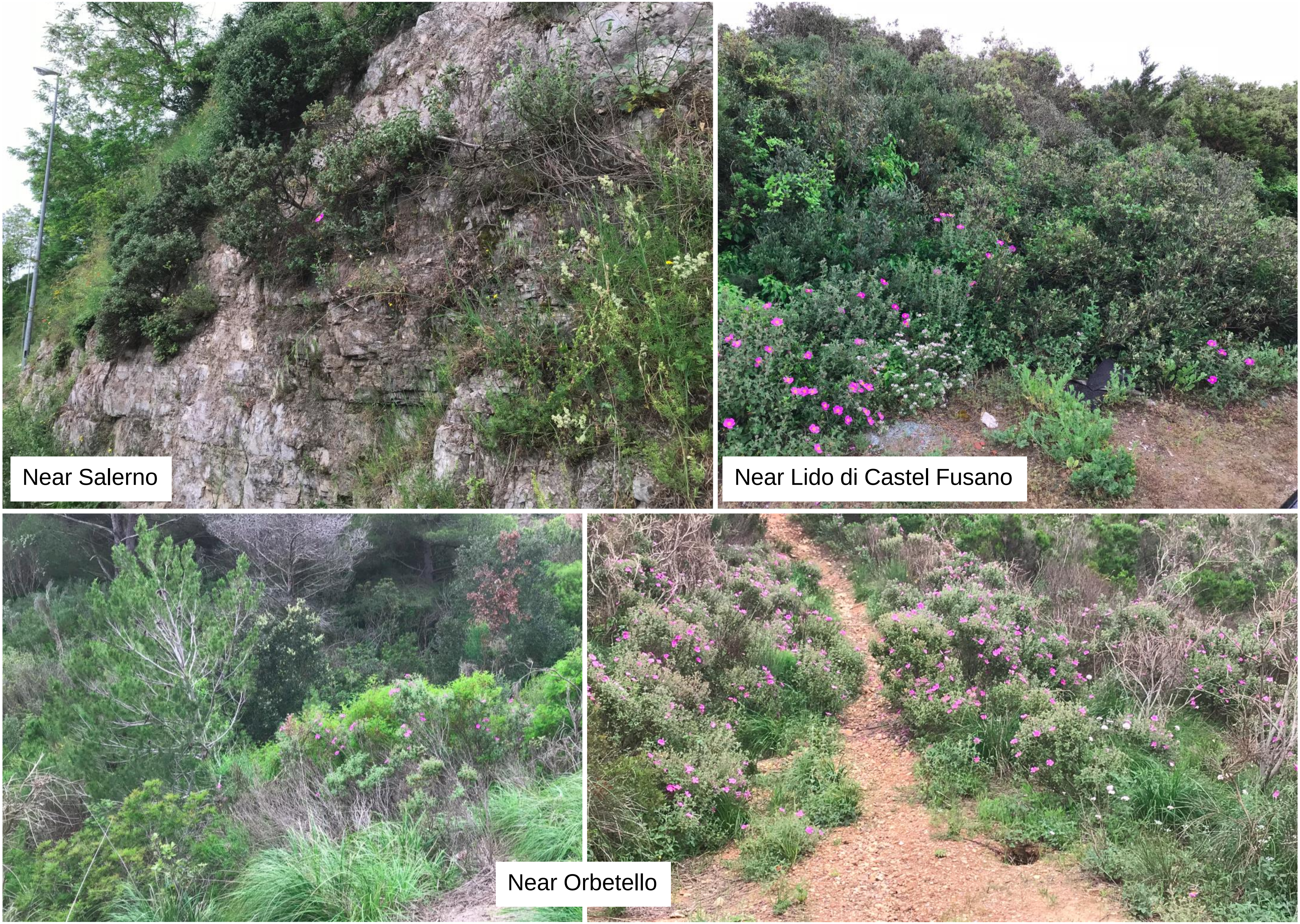

CR1, *C. creticus*, Croatia

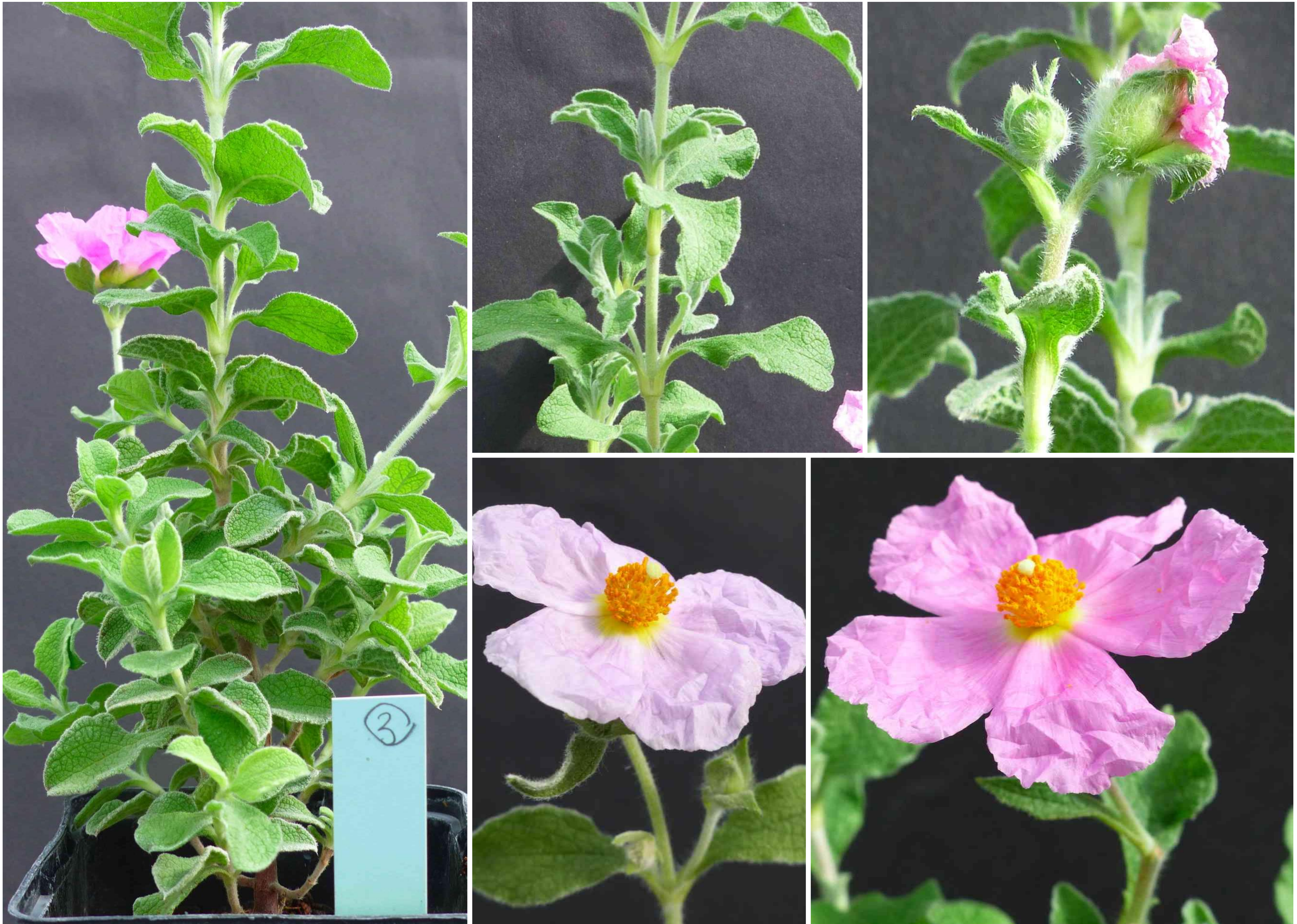

C. creticus, wild populations in Croatia

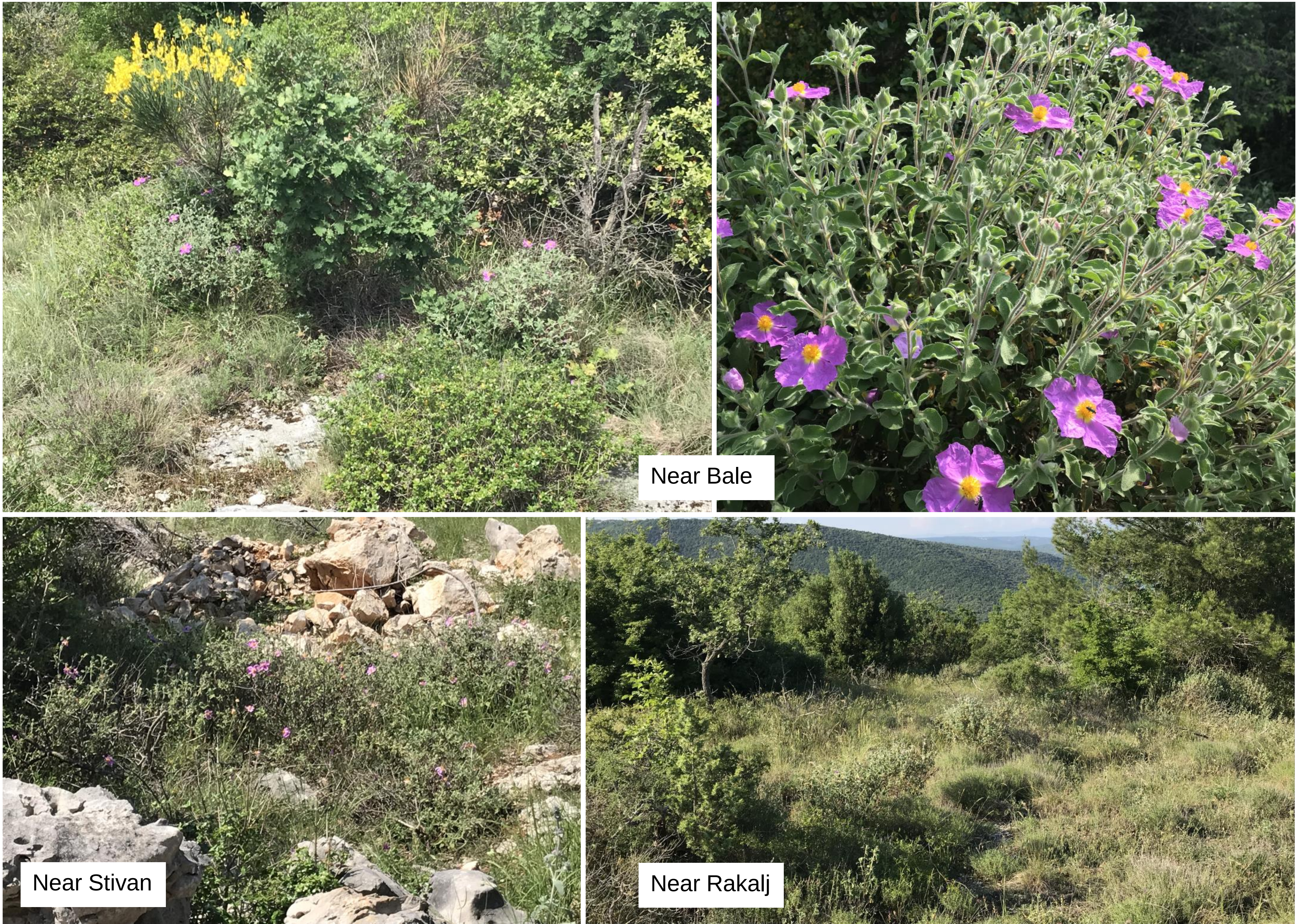

C. creticus, wild populations in Croatia

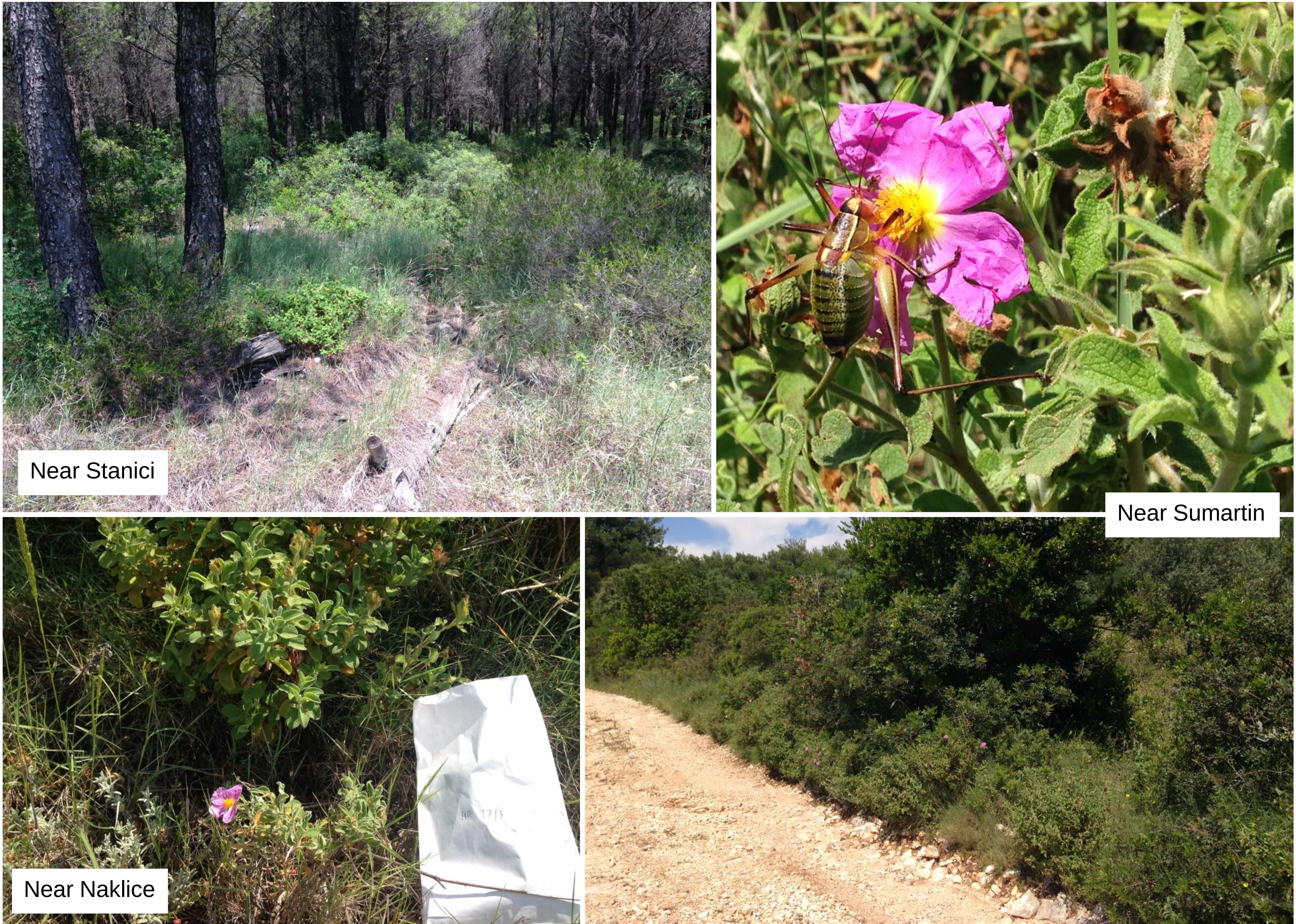

SC1, *C. creticus*, Albania, intra-population variability of flower color

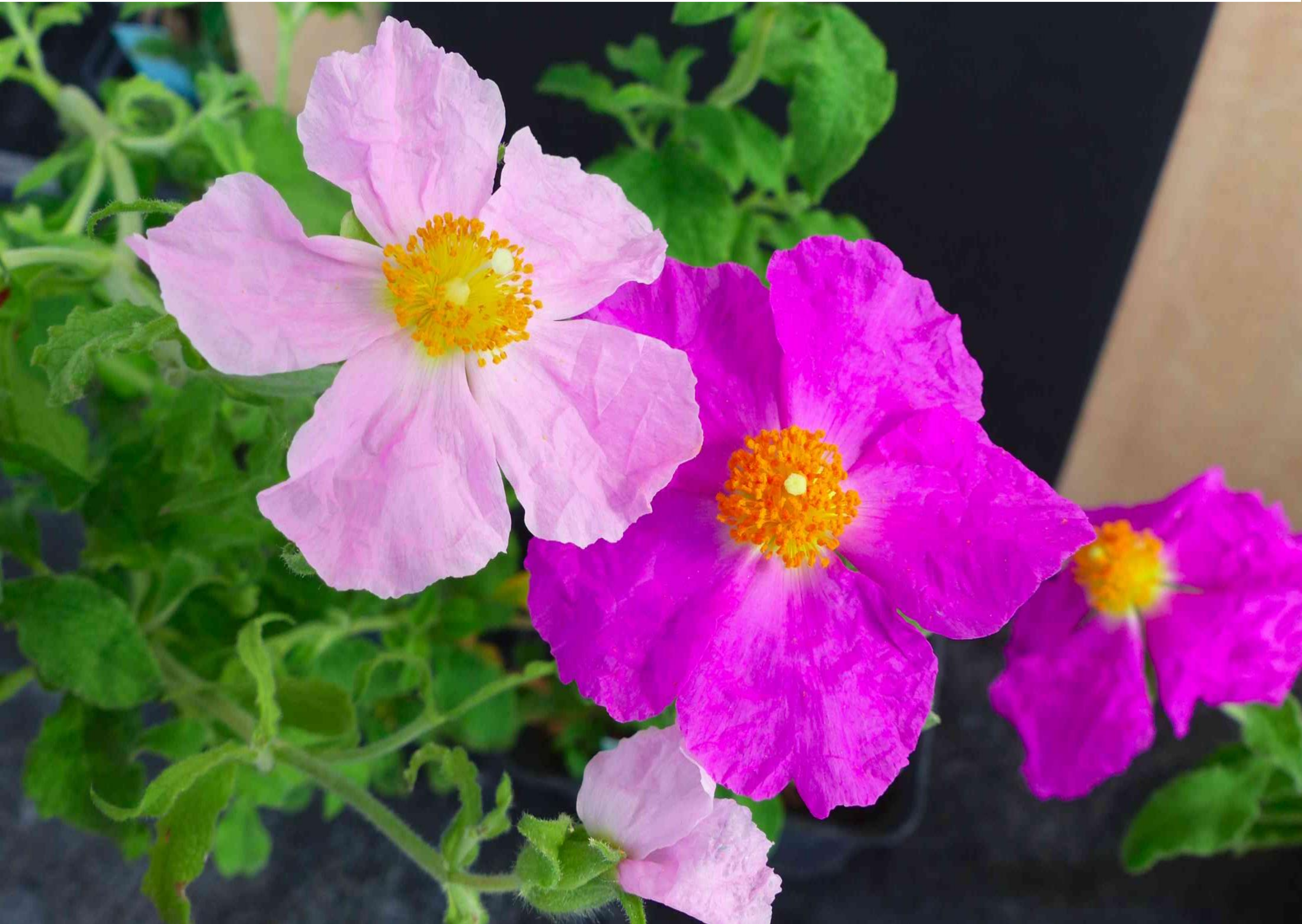

SC2, *C. creticus*, Albania

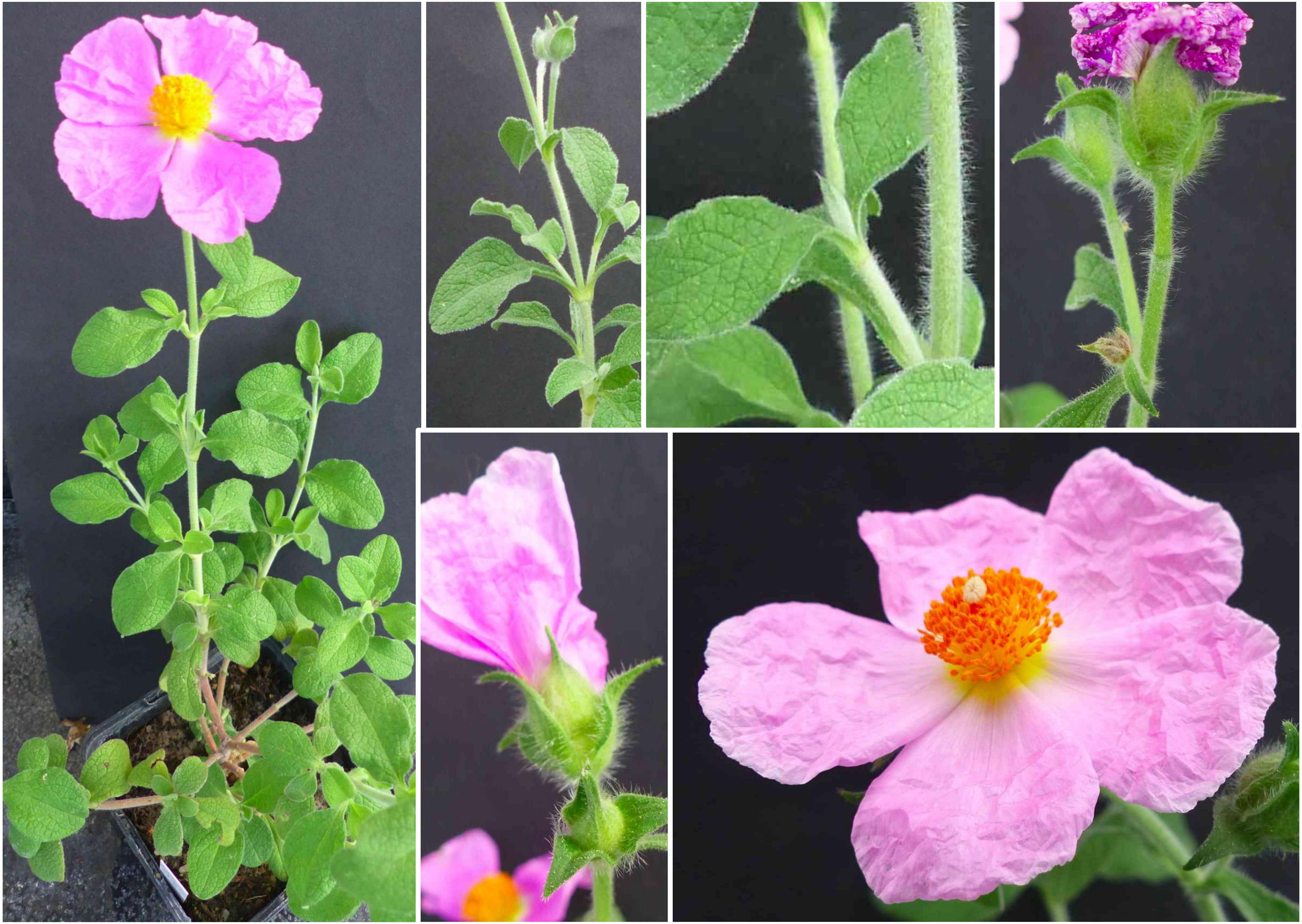

C. creticus, wild populations in Albania

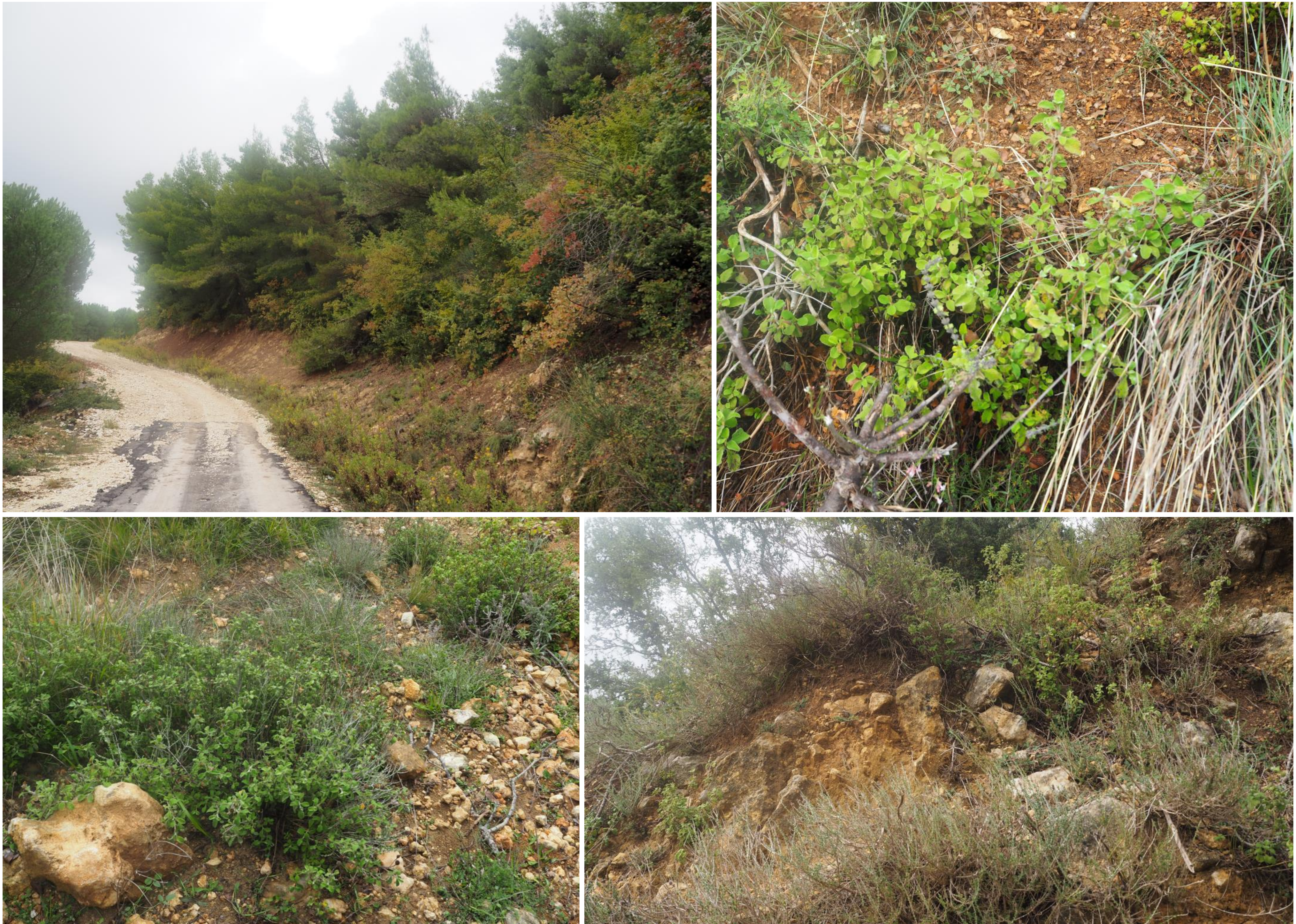

SC21, *C. creticus*, Greece

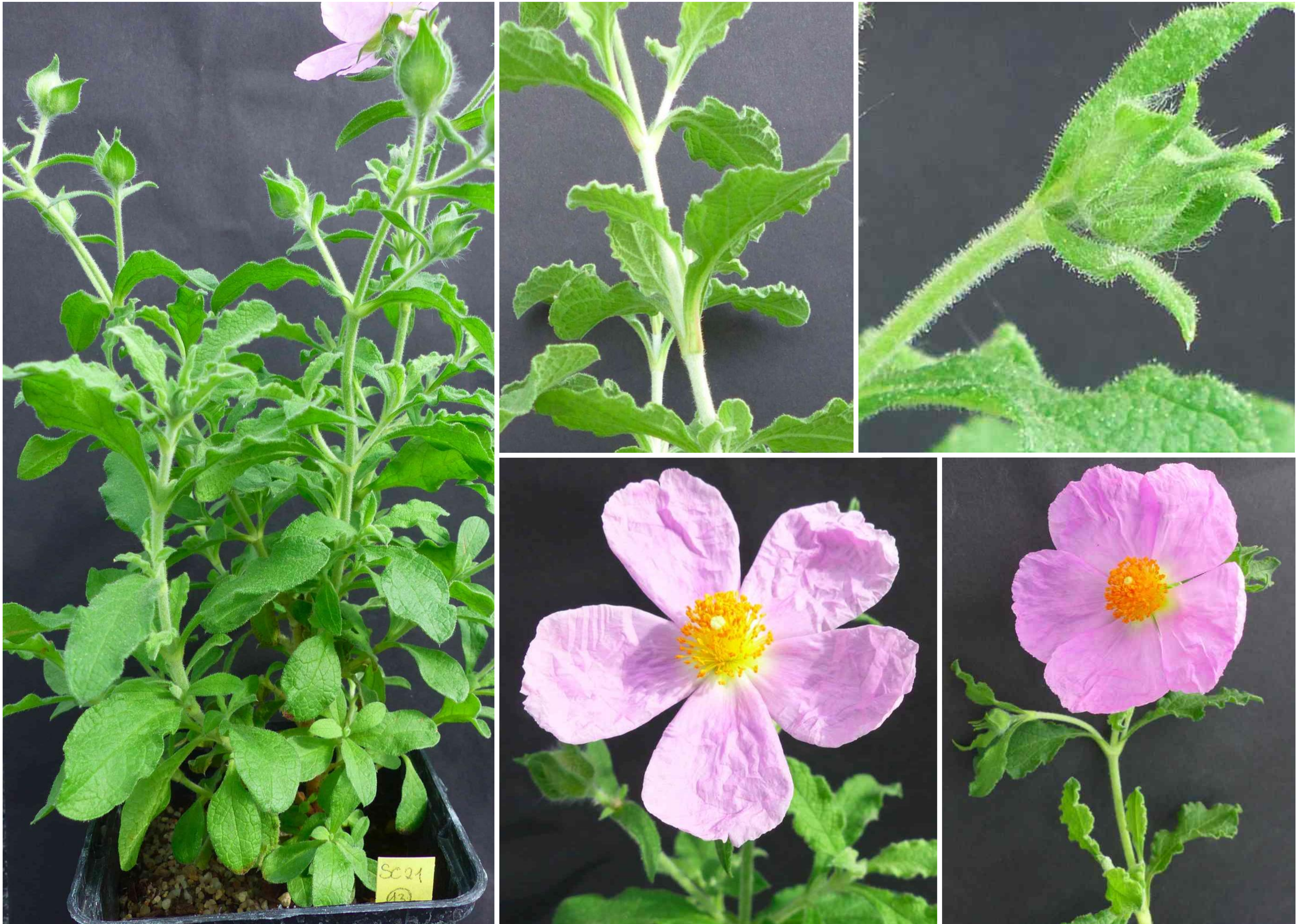

SC22, *C. creticus*, Greece

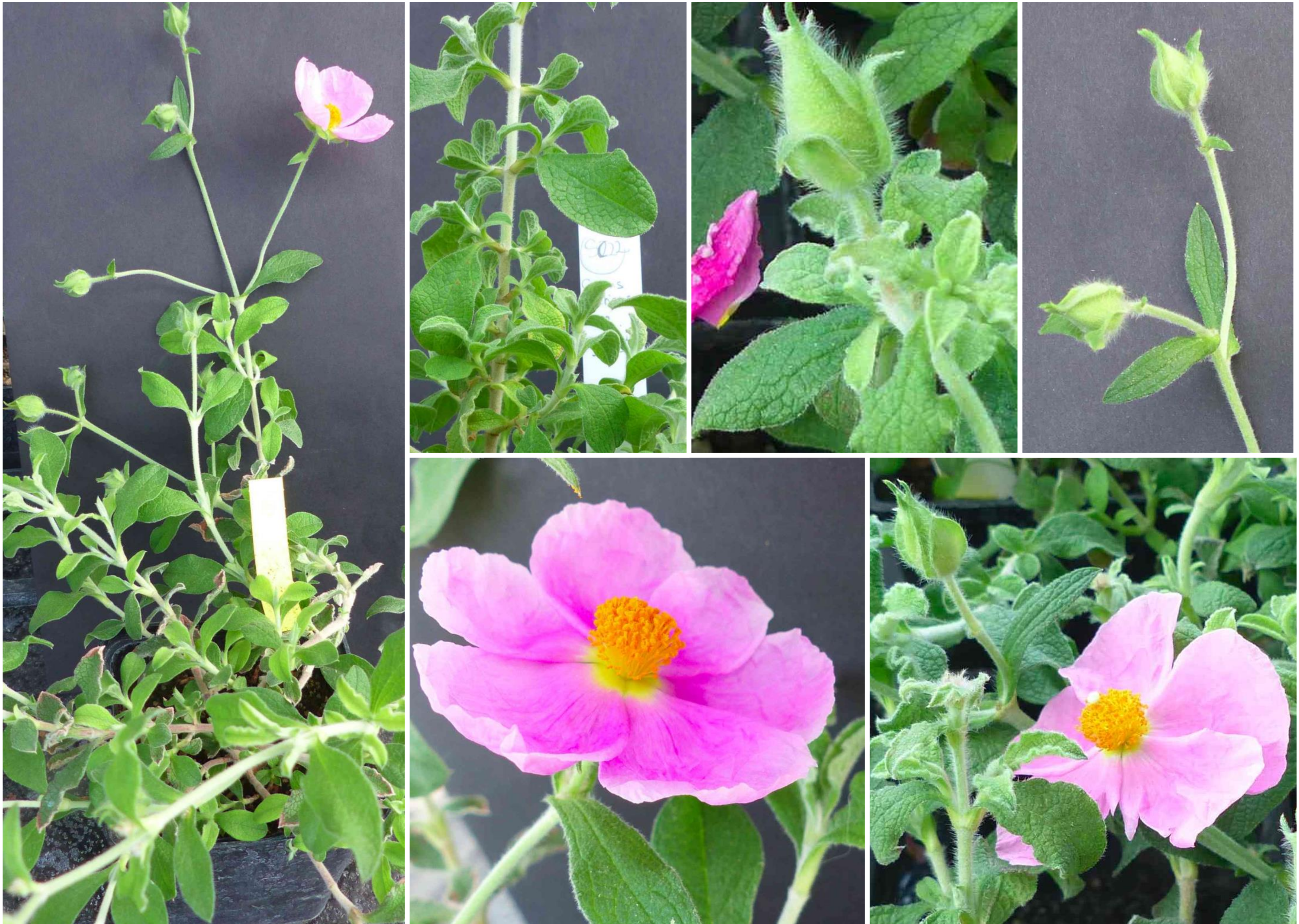

SC19, *C. creticus*, Cyprus

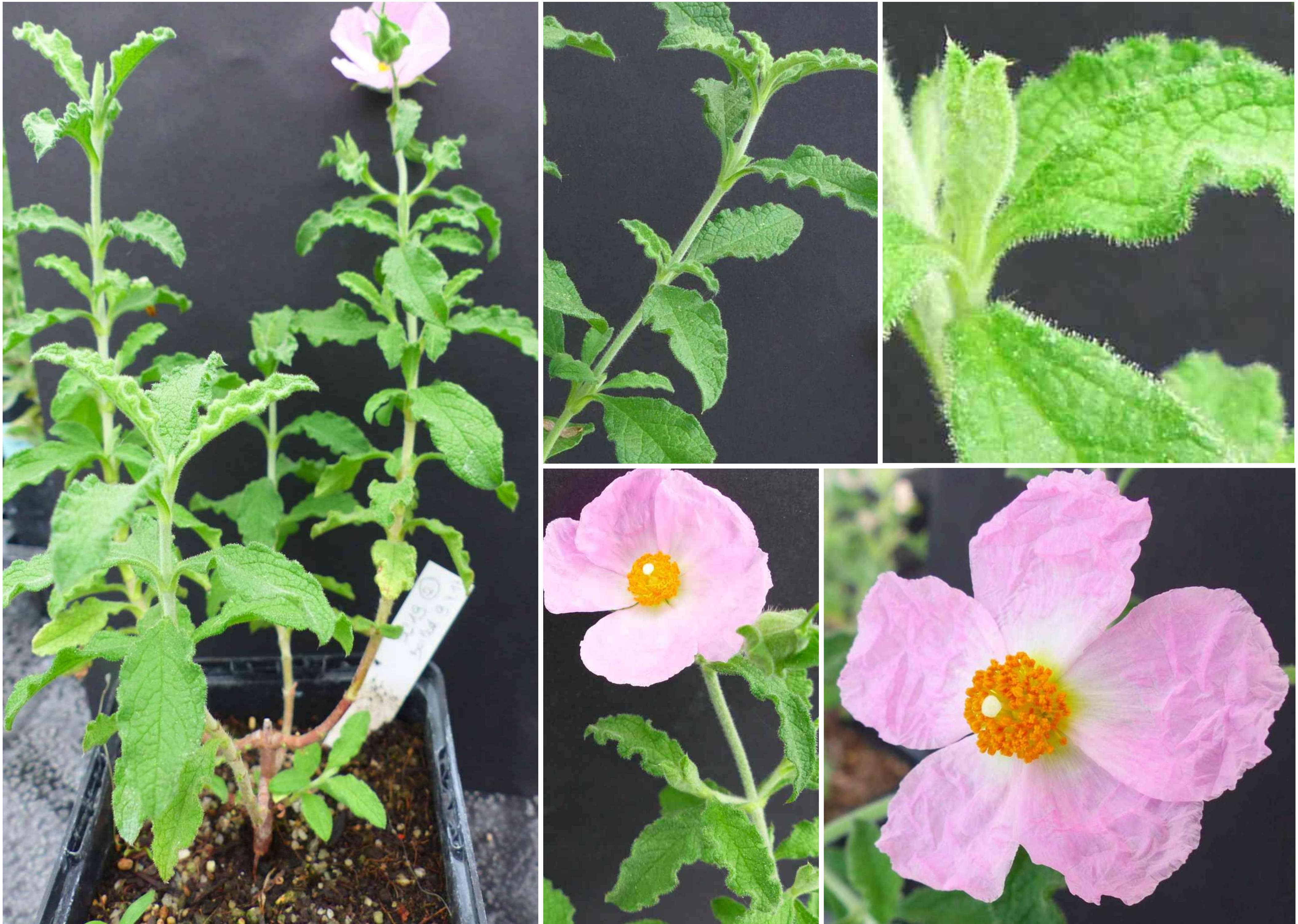

C. creticus, wild populations in Cyprus

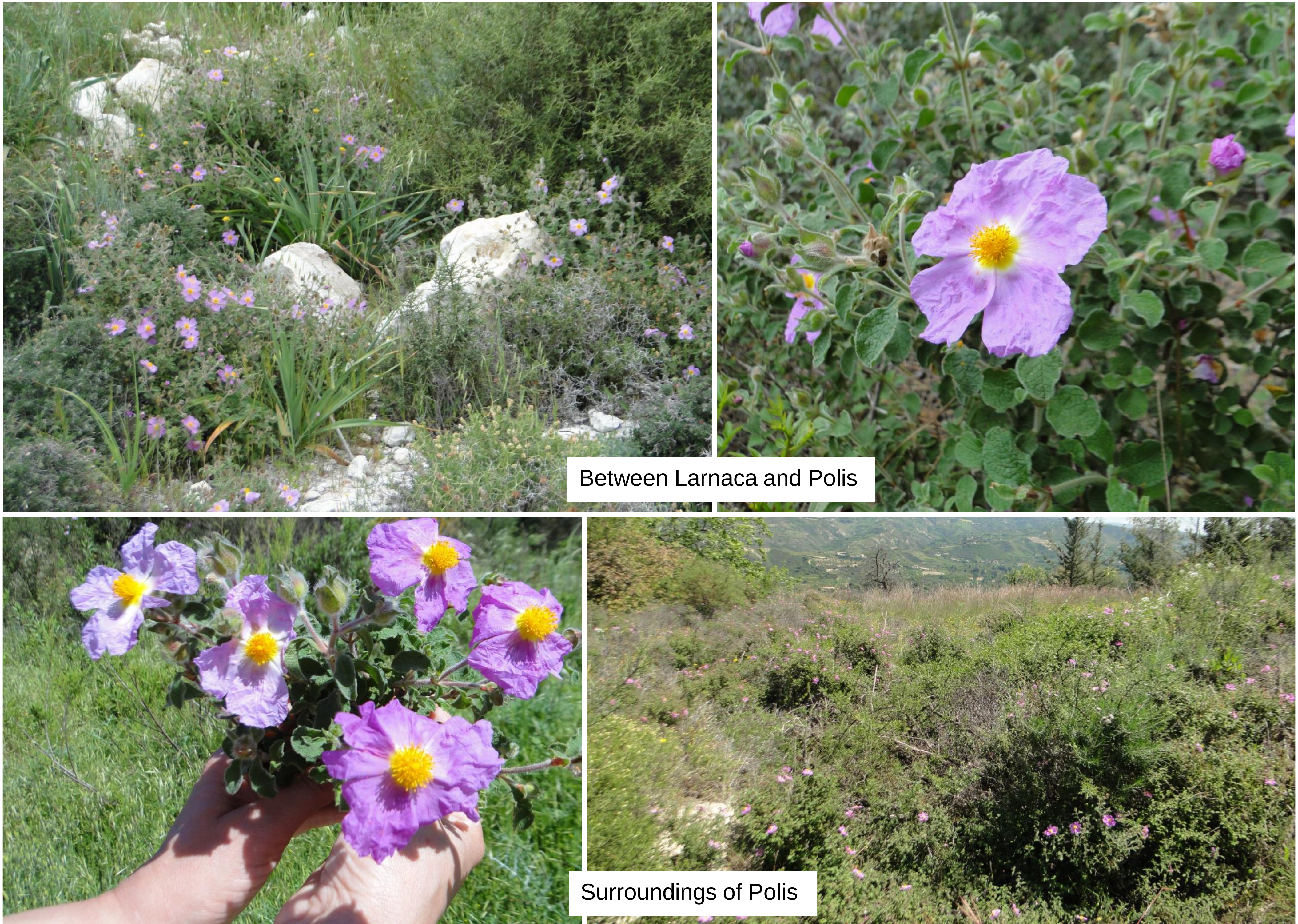

Between Larnaca and Polis

Surroundings of Polis

C. creticus, wild populations in Cyprus

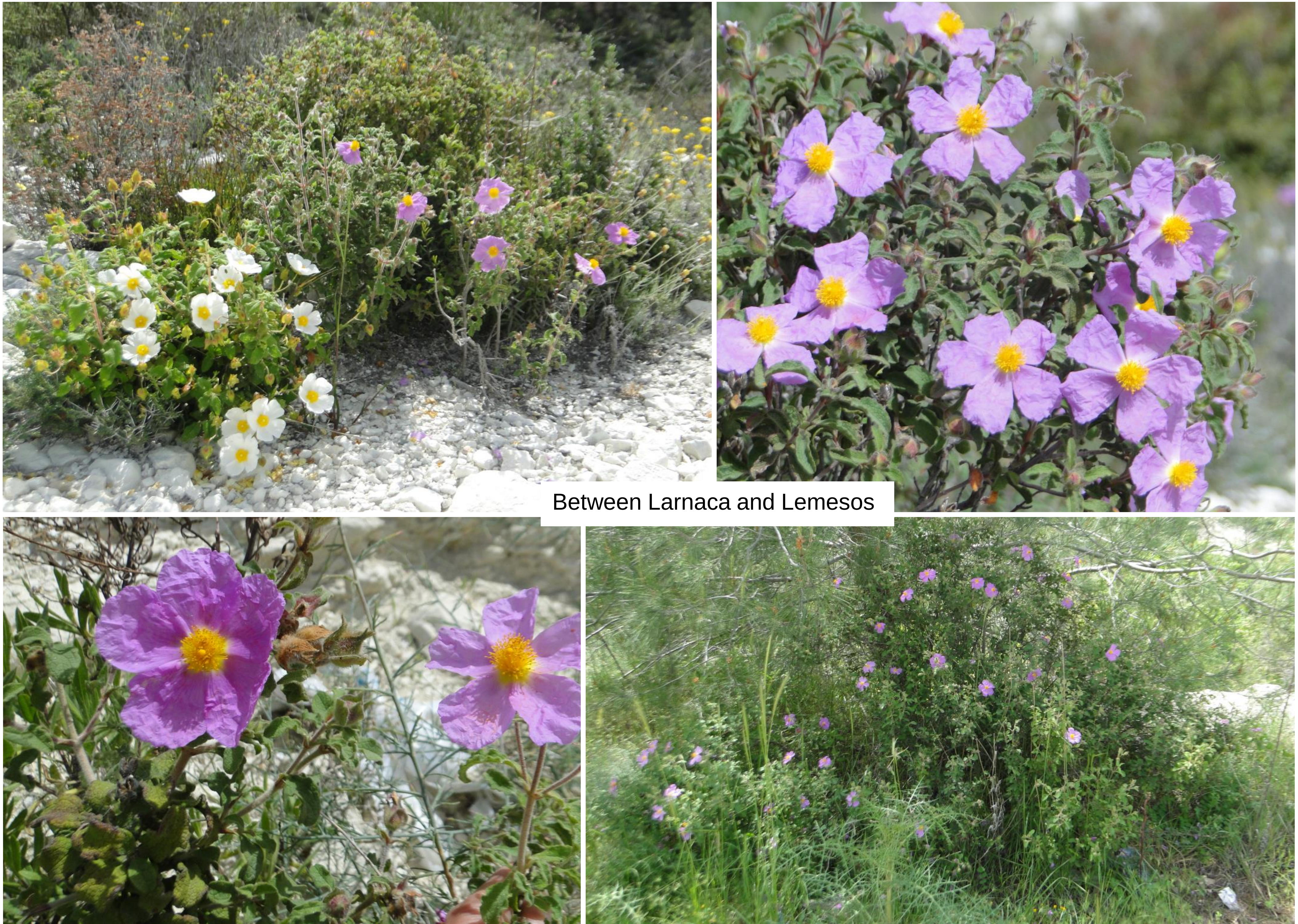

Between Larnaca and Lemesos

SC12, *C. creticus*, Lebanon

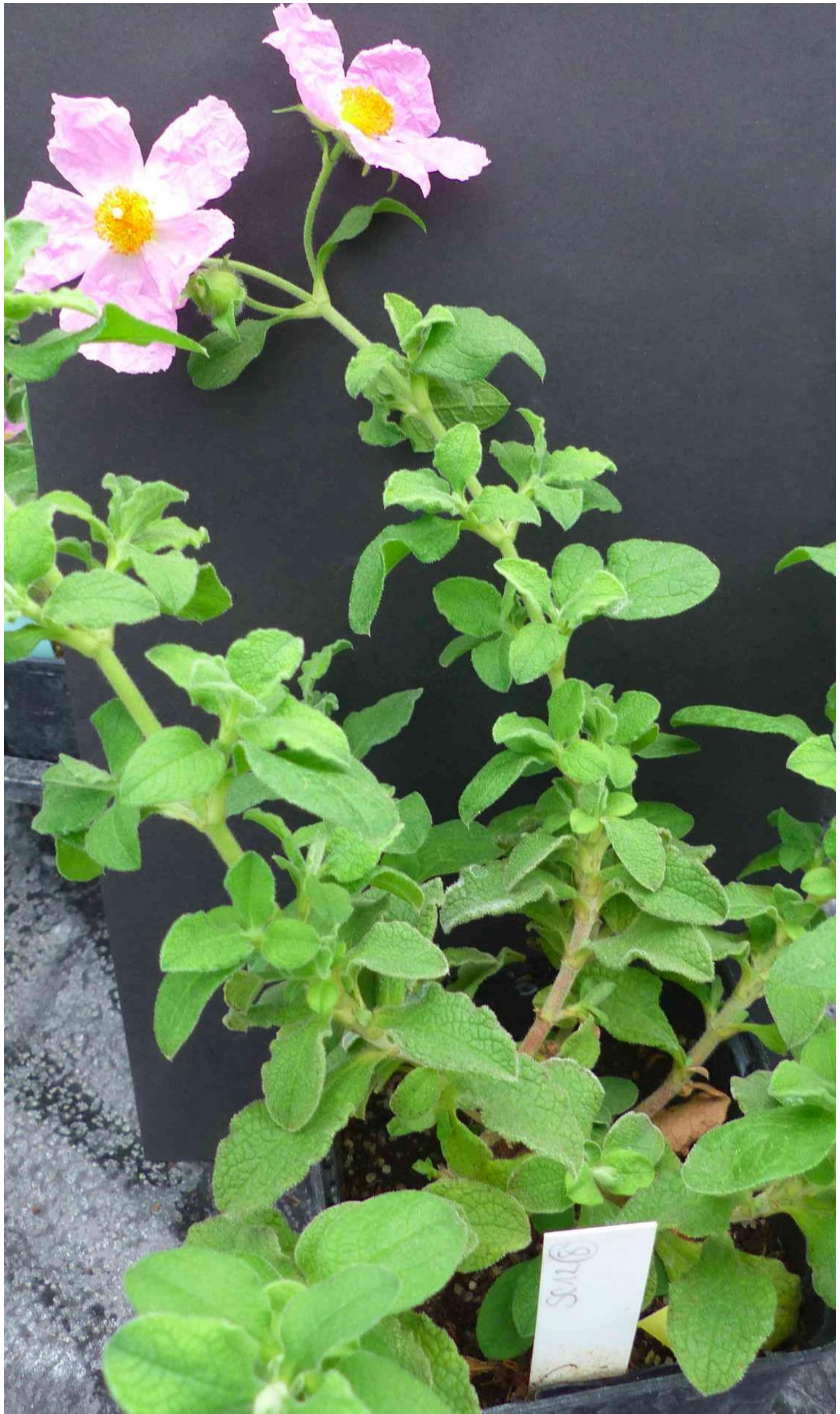

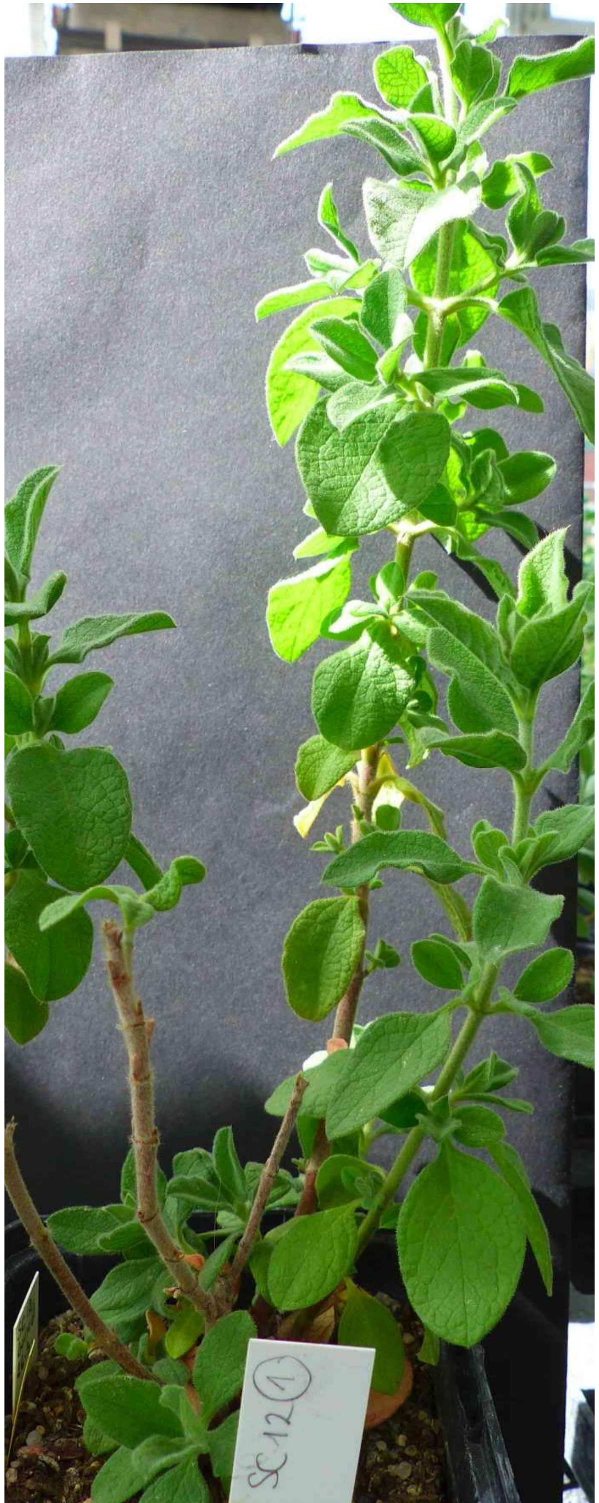

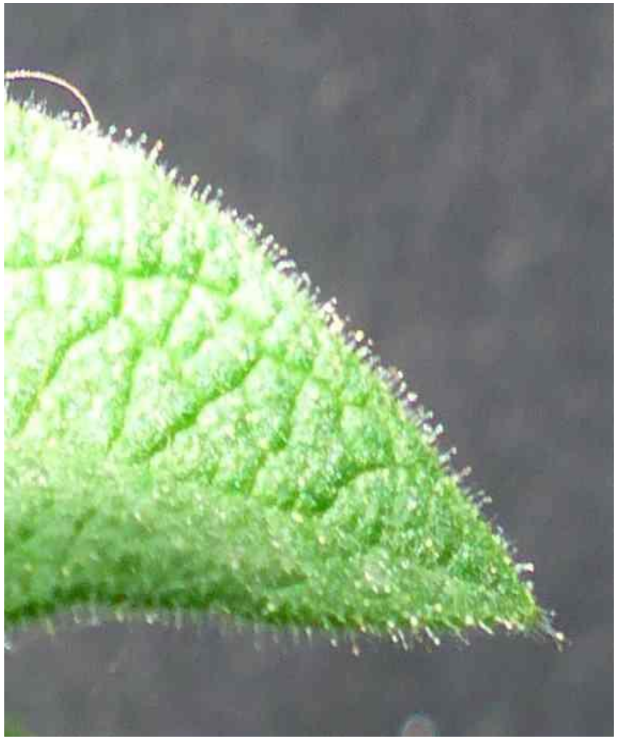

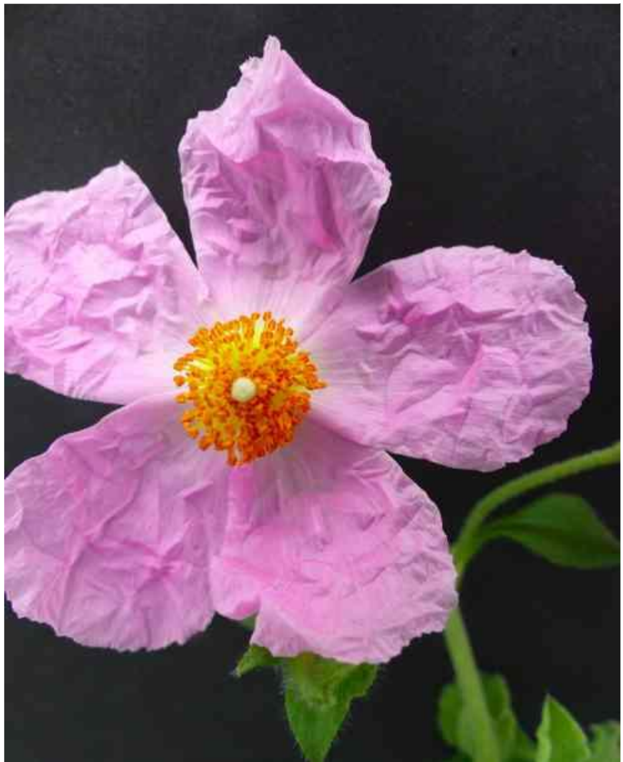

Supplement: Supplementary file 1 [file plants-10-00615-s001.zip › supplementary plants-1150493/PictureGallery.pdf]
