# Supplementary material for: Polyphenol Diversity and Antioxidant Activity of European Cistus creticus L. (Cistaceae) Compared to Six Further, Partly Sympatric Cistus Species
Source: Plants (Basel). 2021 Mar 24;10(4):615. doi: 10.3390/plants10040615 (PMC8063833; doi:10.3390/plants10040615)
Supplement: Supplementary file 1 [file plants-10-00615-s001.zip › supplementary plants-1150493/supplementary plants-1150493.docx]

**Supplementary Materials**


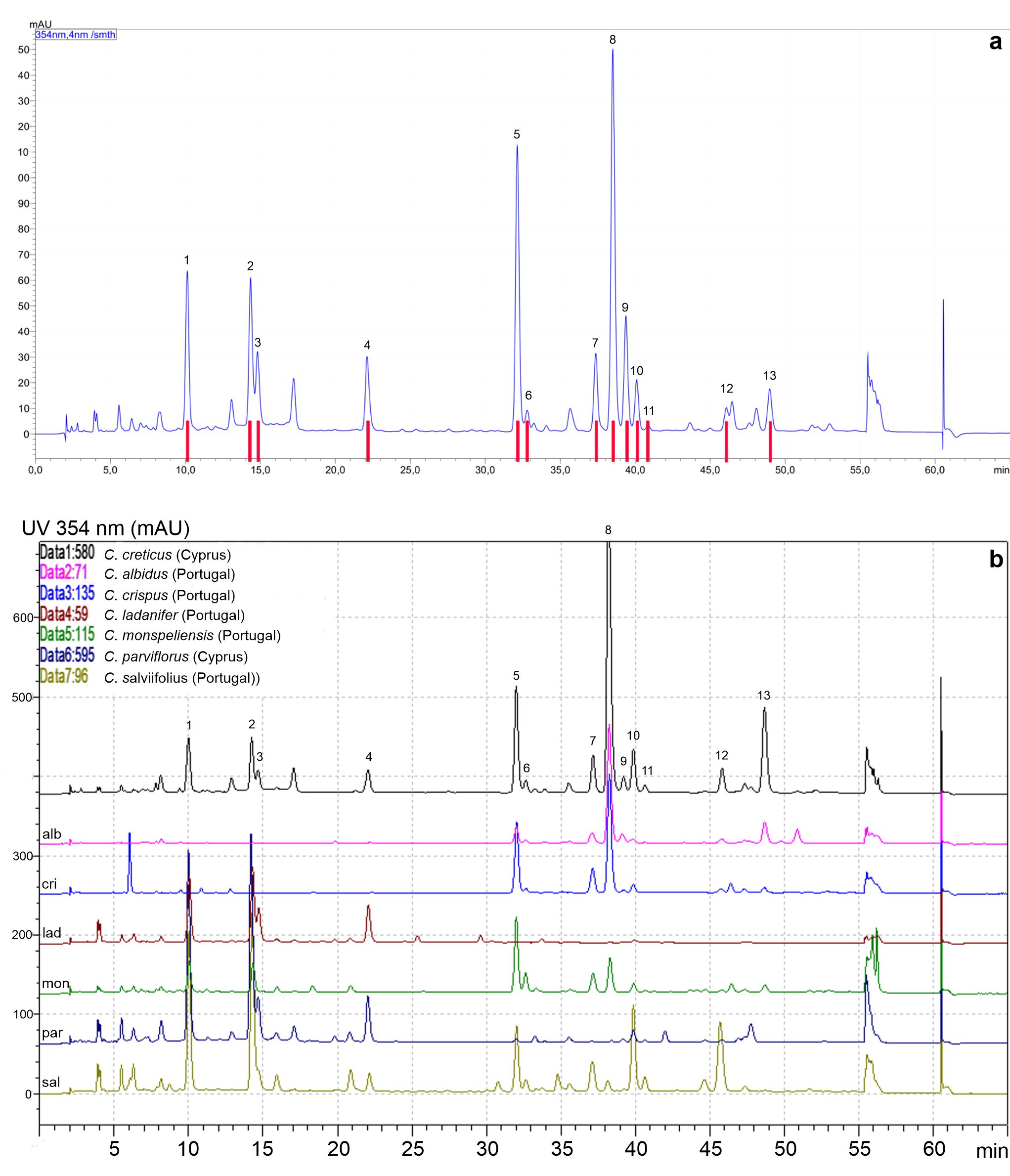
Figure S1: (a) Example chromatogram of Cypriot *C. creticus* recorded at 354 nm*.* Red marks refer to the retention times of the 13 peaks defined after initial inspection of representative chromatograms. The peak numbers indicated in the graph correspond to the peak numbers and compounds listed in Table 1 and Table S1. (b) Direct comparison of representative example chromatograms of *C. creticus* (SRC580, cre), *C. albidus* (SRC71, alb), *C. crispus* (SRC135, cri), *C. ladanifer* (SRC59, lad), *C. monspeliensis* (SRC115, mon), *C. parviflorus* (SRC595, par) and *C. salviifolius* (SRC96, sal). Identification of the quantified main compounds is indicated.


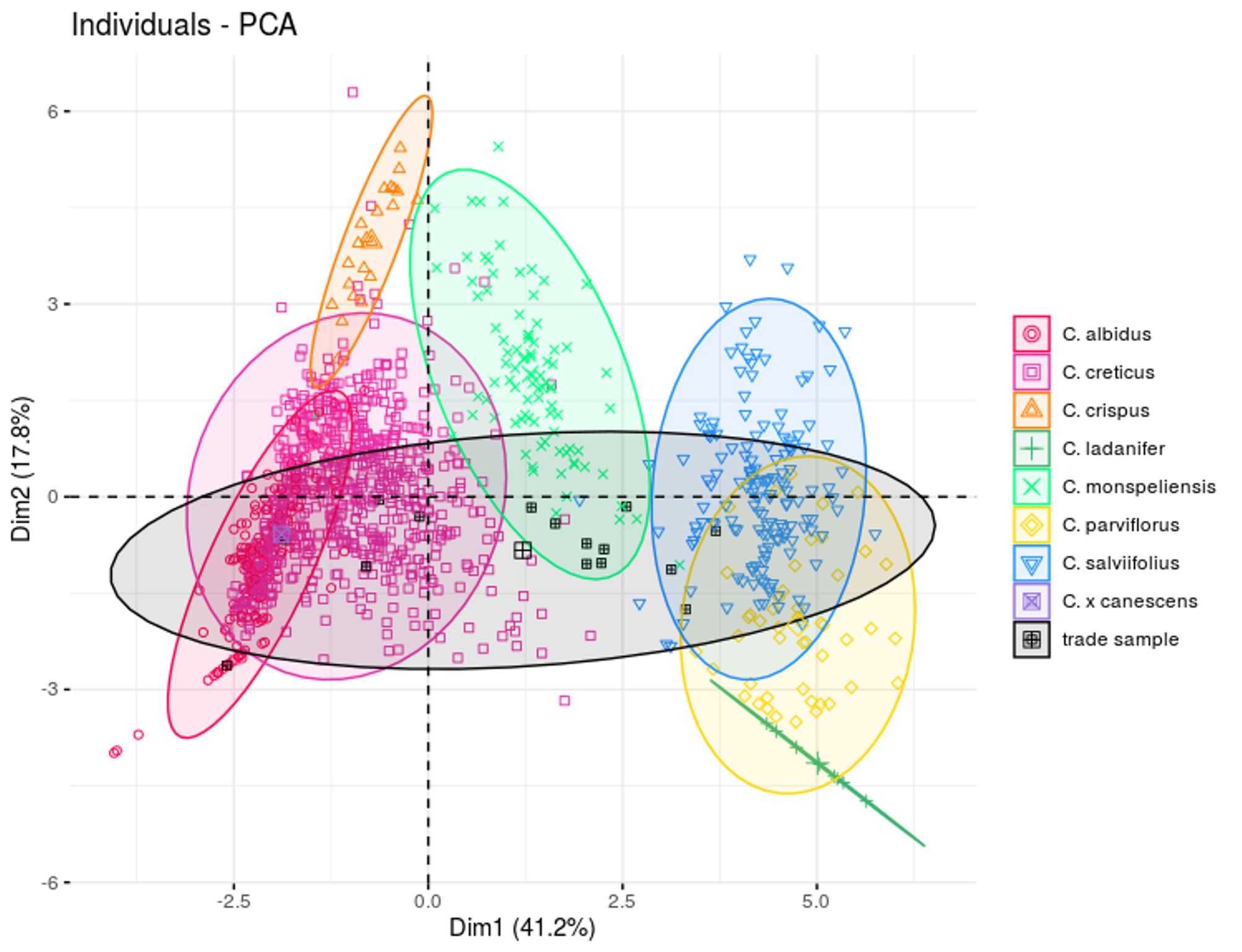


Figure S2: Principal Component Analysis - plot of the first two dimensions from PCA performed over all seven species and the eleven evaluated main compounds (relative area percent). Black squares indicate the position of the 15 trade samples.


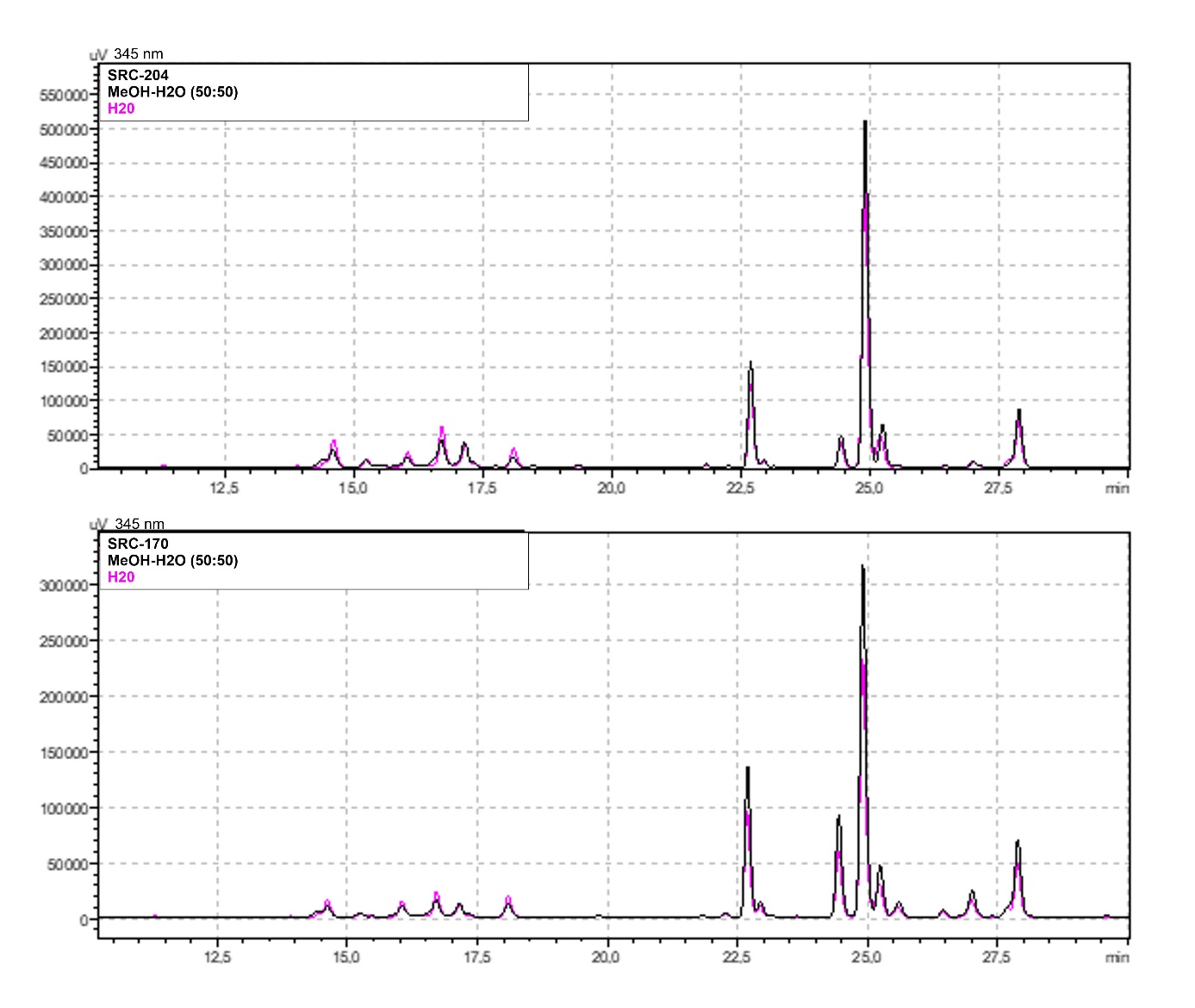


Figure S3: HPLC chromatograms (354 nm) of SRC170 and SRC204 (*C. creticus*, Cyrus), comparison of hydromethanolic extract (50:50; black line) and pure water extract (pink line).

Table S1 (sheet 1): Geographical origin of natural and cultivated Cistus populations, collection details, number of plants analysed, population mean values and standard deviations of quantified main components, total phenolics and antioxidative activity.

Table S1 (sheet 2): Species minimum values, maximum values, mean values and standard deviations of main components, total phenolics and antioxidant activity.

Table S2: Statistical correlation between total phenolics (mg cae/g dry wt), antioxidant activity (mg te/g dry wt) and sums of punicalagin derivatives, myricetin- and quercetin-glycosides (mg/g dry wt).

Picture gallery: View of selected natural *C. creticus* populations and potted *Cistus* plants.
