# Supplementary figures and images for: Polyphenol Diversity and Antioxidant Activity of European Cistus creticus L. (Cistaceae) Compared to Six Further, Partly Sympatric Cistus Species
Source: Plants (Basel). 2021 Mar 24;10(4):615. doi: 10.3390/plants10040615 (PMC8063833; doi:10.3390/plants10040615)

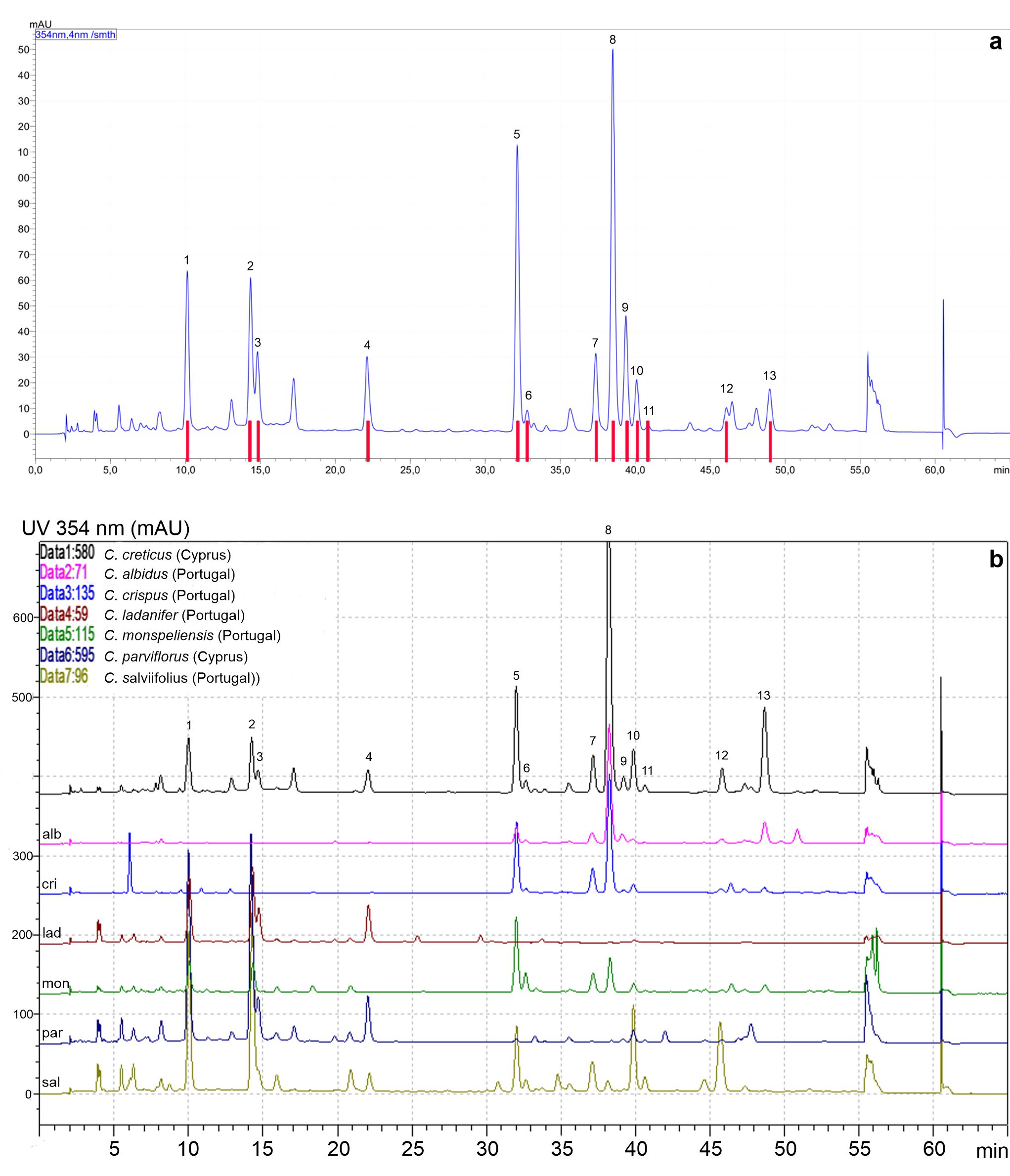

Supplement: Supplementary file 1 [file plants-10-00615-s001.zip › supplementary plants-1150493/FigureS1_Chromatograms.jpg]

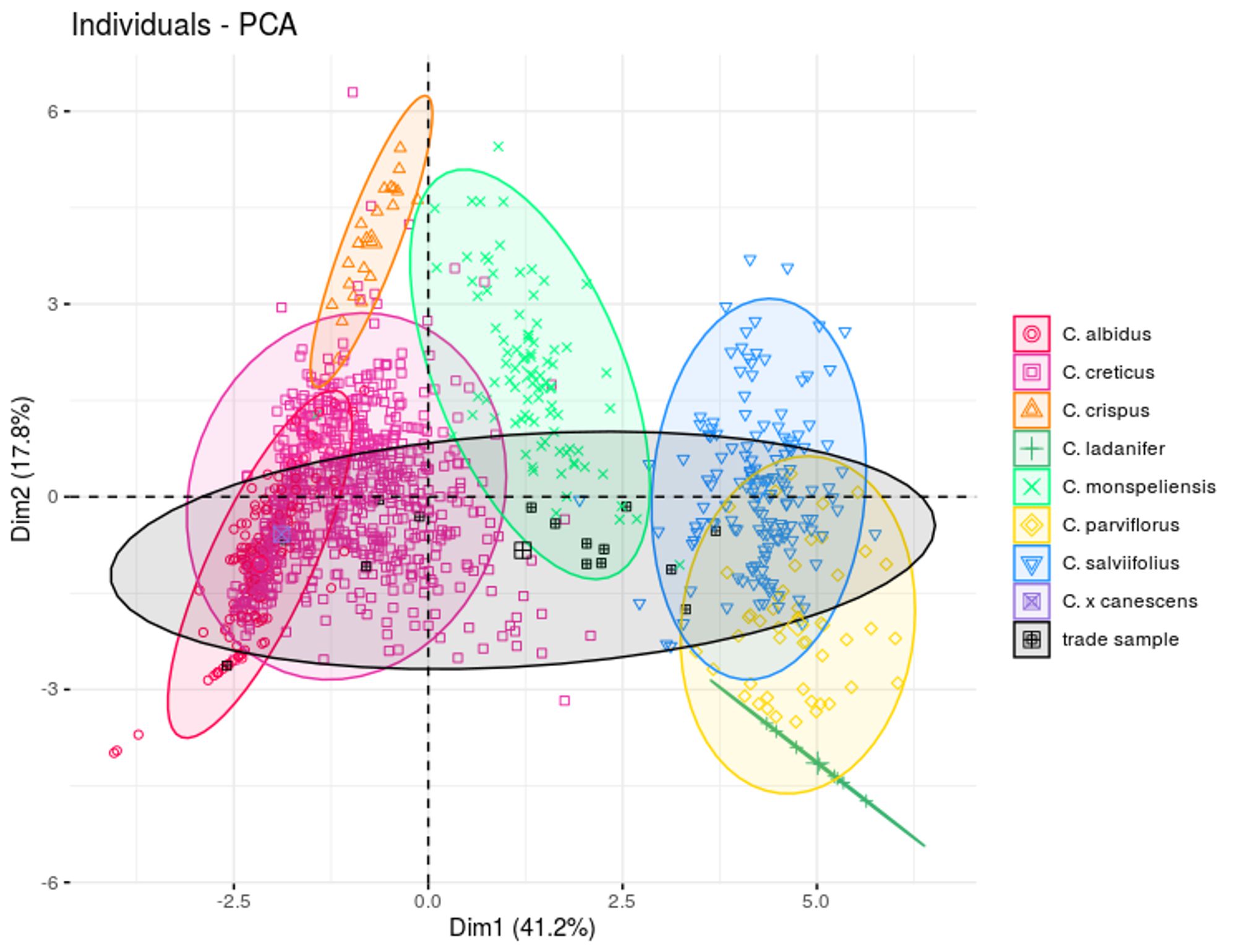

Supplement: Supplementary file 1 [file plants-10-00615-s001.zip › supplementary plants-1150493/FigureS2_PCA_HP.jpg]

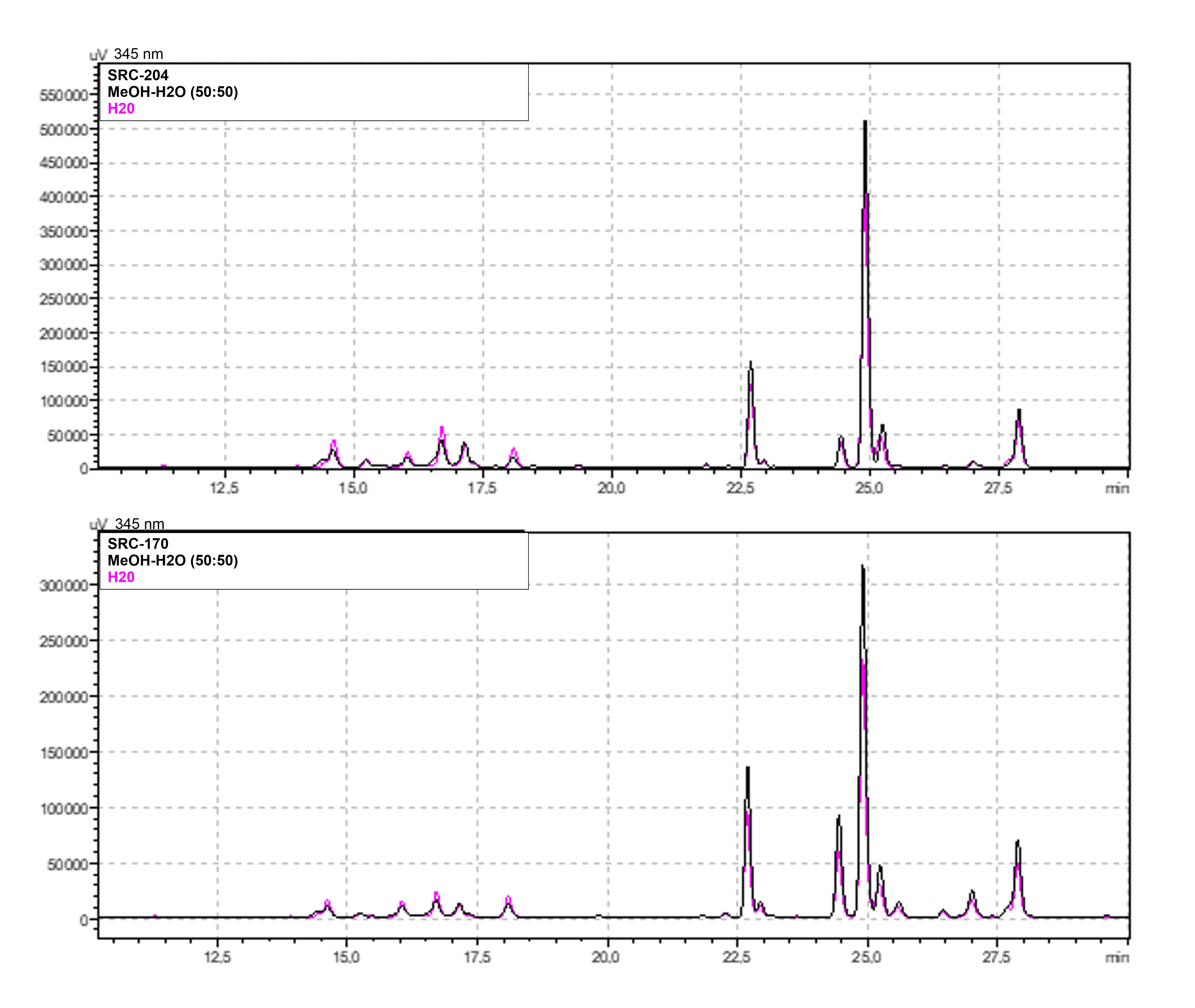

Supplement: Supplementary file 1 [file plants-10-00615-s001.zip › supplementary plants-1150493/FigureS3_MethanolWater.jpg]
